# A Polybasic Plasma Membrane Binding Motif in the I-II Linker Stabilizes Voltage-gated Ca_V_1.2 Calcium Channel Function*

**DOI:** 10.1074/jbc.M115.645671

**Published:** 2015-06-22

**Authors:** Gurjot Kaur, Alexandra Pinggera, Nadine J. Ortner, Andreas Lieb, Martina J. Sinnegger-Brauns, Vladimir Yarov-Yarovoy, Gerald J. Obermair, Bernhard E. Flucher, Jörg Striessnig

**Affiliations:** From the ‡ Institute of Pharmacy, Department of Pharmacology and Toxicology, and Center for Molecular Biosciences, University of Innsbruck, A-6020 Innsbruck, Austria,; the § Department of Physiology and Membrane Biology, UC Davis School of Medicine, Davis, California 95616, and; the ¶ Division of Physiology, Medical University of Innsbruck, A-6020 Innsbruck, Austria

**Keywords:** calcium channel, electrophysiology, phospholipid, plasma membrane, protein motif, Cav1.2, L-type calcium channels, cytoplasmic domain

## Abstract

L-type voltage-gated Ca^2+^ channels (LTCCs) regulate many physiological functions like muscle contraction, hormone secretion, gene expression, and neuronal excitability. Their activity is strictly controlled by various molecular mechanisms. The pore-forming α_1_-subunit comprises four repeated domains (I–IV), each connected via an intracellular linker. Here we identified a polybasic plasma membrane binding motif, consisting of four arginines, within the I-II linker of all LTCCs. The primary structure of this motif is similar to polybasic clusters known to interact with polyphosphoinositides identified in other ion channels. We used *de novo* molecular modeling to predict the conformation of this polybasic motif, immunofluorescence microscopy and live cell imaging to investigate the interaction with the plasma membrane, and electrophysiology to study its role for Ca_v_1.2 channel function. According to our models, this polybasic motif of the I-II linker forms a straight α-helix, with the positive charges facing the lipid phosphates of the inner leaflet of the plasma membrane. Membrane binding of the I-II linker could be reversed after phospholipase C activation, causing polyphosphoinositide breakdown, and was accelerated by elevated intracellular Ca^2+^ levels. This indicates the involvement of negatively charged phospholipids in the plasma membrane targeting of the linker. Neutralization of four arginine residues eliminated plasma membrane binding. Patch clamp recordings revealed facilitated opening of Ca_v_1.2 channels containing these mutations, weaker inhibition by phospholipase C activation, and reduced expression of channels (as quantified by ON-gating charge) at the plasma membrane. Our data provide new evidence for a membrane binding motif within the I-II linker of LTCC α_1_-subunits essential for stabilizing normal Ca^2+^ channel function.

## Teaser

**Background:** L-type Ca^2+^ channels (LTCCs) are fine-tuned by different molecular mechanisms.

**Results:** Pore-forming α_1_-subunits of LTCCs contain a polybasic amino acid sequence within their I-II linkers that binds to the plasma membrane. This polybasic motif is required for normal channel gating and modulation.

**Conclusion:** The polybasic cluster stabilizes normal channel activity.

**Significance:** We discovered a new modulatory domain of LTCCs within their pore-forming α_1_-subunit.

## Introduction

Ca^2+^ influx through voltage-gated L-type Ca^2+^ channels (LTCCs)^3^ is essential for many cellular events. It causes muscle contraction, initiates hormone secretion and neurotransmitter release, tunes neuronal excitability, and regulates gene expression (1, 2). LTCCs are large multiprotein complexes consisting of a pore-forming transmembrane α_1_-subunit and accessory intracellular β- and extracellular α_2_δ-subunits. To adapt Ca^2+^ entry to different cellular needs, functional diversity is achieved by multiple molecular mechanisms. Four different α_1_-subunits (Ca_V_1.1–1.4) can confer different biophysical properties (1) that are further adjusted by alternative splicing (3–5). In addition, the channels' accessory subunits tune their gating behavior and various protein interaction partners further provide LTCC currents with cell- and tissue-specific properties (1). However, rapid regulatory changes in current dynamics cannot be accomplished by changing channel composition. Instead, quick adaptive responses of channel activity require fast regulatory processes, including Ca^2+^ entry itself (inducing inactivation through channel-bound calmodulin (6)), phosphorylation by various kinases (7–9), extracellular pH (10), and direct G-protein modulation (11). Next to these mechanisms, membrane lipids are also important regulators of Ca^2+^ channel activity (12, 13). Depletion of phosphatidylinositol 4,5-bisphosphate (PIP_2_) from the plasma membrane causes a rapid decrease of Ca^2+^ channel activity. In excised membrane patches, non-LTCC currents (Ca_V_2.1 and Ca_V_2.2) run down within minutes, which is attenuated by application of PIP_2_ or its water-soluble analogue diC8-PIP_2_ (14, 15). Rapid depletion of membrane PIP_2_ content in intact cells by muscarinic 1 (M1) receptor activation, voltage-dependent phosphatases, or rapamycin-induced translocation of inositol-lipid phosphatases confirmed the direct dependence of Ca_V_1 (Ca_V_1.2 and Ca_V_1.3) and Ca_V_2 (Ca_V_2.1 and Ca_V_2.2) channels on endogenous membrane PIP_2_ (16). Channel inhibition by PIP_2_ depletion occurs in a β-subunit-dependent manner because the β_2a_-subunit, an isoform anchored to the plasma membrane by palmitoylation, attenuates inhibition (13, 17). Together with observations that arachidonic acid inhibits channel activity, a model has been proposed in which PIP_2_, the fatty acid side chain of palmitoylated β_2a_-subunits of voltage-gated Ca^2+^ channels (VGCCs), and arachidonic acid have overlapping binding sites, resulting in complex channel regulation by lipid metabolism (12, 17–19). There is also experimental evidence for more than one PIP_2_ regulatory site in VGCCs: a higher affinity stimulatory “S” site supporting channel activity and a lower affinity inhibitory site (“R” site) stabilizing reluctant gating properties (12, 14, 20). Current working models suggest at least one facilitatory PIP_2_ site on LTCCs and at least two on Ca_V_2.2 channels (16).

Anionic phospholipids, like phosphatidylserine and phosphoinositides, target proteins with clusters of positive charges to the plasma membrane (21, 22). Despite unique insights into the structural basis of PIP_2_ modulation within the crystal structure of K^+^ channels (23, 24) and the identification of potential polyphosphoinositide binding domains within several ion channel proteins (for a review, see Refs. 13 and 25), the structural basis for fast modulation of VGCC function by plasma membrane lipids is unknown. It occurs at a very fast time scale, indicating that channel-lipid interactions are rapidly reversible and do not require more complex biochemical pathways, such as channel internalization (26). Similar to K^+^ channels (23, 24), it appears likely that cytoplasmic domains located close to the plasma membrane participate in lipid interactions that stabilize channel function and allow modulation of channels by activation of phospholipase C (PLC). In addition to fast channel modulation, it is at present also unknown whether anionic lipids participate in the constitutive stabilization of VGCC function, which would be expected for very high affinity interactions not subject to regulation by changes in plasmalemmal phospholipid content (27).

Here we report the identification of a polybasic cluster within the cytoplasmic I-II linker of Ca_V_1.2 α_1_-subunits, which specifically binds to the plasma membrane. Activation of PLCs and polyphosphoinositide breakdown together with elevated intracellular Ca^2+^ levels reverse membrane binding. Four positively charged arginines are required for binding, most likely by strongly favoring a straight helix conformation of that region, positioning it entirely at the interface between the hydrocarbon core and lipid headgroups. Their neutralization in the intact channel facilitates channel opening and thus suggests a role of this polybasic cluster in stabilizing normal channel activity.

## Experimental Procedures

All chemicals, reagents and antibodies were purchased from Sigma-Aldrich (Vienna, Austria) except where otherwise indicated. diC8-PIP_2_ was purchased from Echelon Biosciences Inc. (Salt Lake City, UT).

### 

#### 

##### Cloning of cDNA Constructs

The Ca_V_ α_1_-subunits used in this study were identical with GenBank^TM^ sequence accession numbers X05921 (Ca_V_1.1 α_1_), X15539 (Ca_V_1.2 α_1_), EU363339 (Ca_V_1.3 α_1_), and AJ224874 (Ca_V_1.4 α_1_).

For immunofluorescence experiments, Ca_V_1 α_1_ I-II linkers (amino acid numbering according to respective accession number: Ca_V_1.1, 335–432; Ca_V_1.2, 436–554; Ca_V_1.3, 407–523; Ca_V_1.4, 373–518) were cloned into vector pCIneo (Promega, E1841) in fusion with a FLAG tag at the C terminus. Ca_V_1.2 I-II linker mutations were cloned into vector p3XFLAG-CMV-10 with a Myc tag at the C terminus and with a triple FLAG tag at the N terminus. The C terminus of Ca_V_1.3 and the Ca_V_1.3 W441A-I-II linker mutant were also cloned into vector p3XFLAG-CMV-10. Ca_V_1.2 I-II linker mutations 4R4A and 4R4E were generated by splicing by overlap extension PCR (28) and cloned into vector mRFP-C1 with mRFP at the N terminus. Vector mRFP-C1 was generated by amplifying mRFP sequence from pCX-mRFP1 vector (29) to replace EYFP in pEYFP-C1 vector (Clontech, 6005-1). The C-terminally V5-tagged β-subunit β_2a_ (M80545), _C3S/C4S_β_2a_, and β_3_ (NM_012828) constructs were expressed as described previously (30, 31).

The generation of N-terminally GFP-tagged β_3_ (expression vector pCI-neo (Promega), generously provided by Manfred Grabner (32)) and C-terminally EGFP-tagged β_2a_-subunits (expression vector pbA (33)) was described recently. mRFP-tagged pleckstrin homology (PH) domain of PLCδ (PH-PLCδ) was kindly provided by the Netherlands Cancer Institute (34). Untagged M1 receptor was generated from cyan fluorescent protein-labeled M1 receptor construct (generously provided by Stefan Böhm (Medical University of Vienna, Austria)) by restriction digestion and cloning into vector pCIneo. Peptide 526–554 with an N-terminal GFP tag (_GFP_526–554) was generated by PCR cloning into vector EGFP (35). Lyn_11_-FRB, mRFP-FKBP-pseudojanin, and GFP-PH-Osh2x2 constructs were kindly provided by Dominik Oliver and Gerald Hammond (36, 37). α_1_-Subunit mutants Ca_V_1.2L_4A_ and Ca_V_1.2L_4E_ were generated by splicing by overlap extension-PCR and cloned into vector pCIneo. For short Ca_V_1.2 α_1_ mutations, Ca_V_1.2S_4A_ and Ca_V_1.2S_4E_, a stop codon was generated by PCR at position 1800 in the Ca_V_1.2 α_1_-subunit in pCIneo. The integrity of all constructs was confirmed by DNA sequencing (Microsynth, Eurofins).

##### Cell Culture and Transfection

For immunofluorescence microscopy, live cell imaging (LCI), and electrophysiology, tsA-201 cells were cultured in Dulbecco's modified Eagle's medium (DMEM), 10% fetal calf serum (Gibco, 10500.064), 2 mm glutamine (Sigma, G753) penicillin (10 units/ml), and streptomycin (10 μg/ml) and maintained at 37 °C in a humidified environment with 5% CO_2_. Cells were grown and split when they reached about 80% confluence using 0.05% trypsin for cell dissociation. Cells were transiently transfected using Ca^2+^-phosphate precipitation as described previously (38). For immunofluorescence and LCI, cells were replated 24 h after transfection onto 12 mm (for immunofluorescence) and 18 mm (for LCI) poly-l-lysine-coated coverslips and kept at 37 °C for 24–48 h until further experimentation. For whole-cell patch clamp recordings, tsA-201 cells were transiently transfected with equimolar ratios of cDNA encoding C-terminally long or short wild-type or mutant Ca_V_1.2 α_1_-subunits together with auxiliary β_3_- (rat, NM_012828) and α_2_δ_1_- (rabbit, NM_001082276) subunits. To visualize transfected cells, GFP was co-transfected. Cells were then plated onto a 35-mm culture dish coated with poly-l-lysine. The cells were kept at 30 °C and 5% CO_2_ and subjected to electrophysiological measurements about 48–72 h after transfection.

##### Immunofluorescence Microscopy

48 h after plating, tsA-201 cells were fixed in 4% (w/v) paraformaldehyde (Electron Microscopy Sciences, 15710) for 15 min at room temperature. Cells were washed thoroughly with phosphate-buffered saline and blocked for 30 min at room temperature with 5% (w/v) normal goat serum (GibcoBRL, 16210-064) in 0.2% (v/v) Triton/phosphate-buffered saline for cell permeabilization. Cells were incubated overnight at 4 °C with primary antibodies diluted in washing buffer containing 0.2% (v/v) Triton X-100 and 0.2% (w/v) BSA (immunoglobulin-free) in phosphate-buffered saline. After washing the cells in washing buffer, they were incubated with the secondary antibody for 1 h at room temperature in the dark. Washing was repeated, and the coverslips were mounted with Vectashield mounting medium (Vector Laboratories, H-1000) and sealed with nail polish on microscope slides. The following antibodies were employed: mouse monoclonal anti-V5, working dilution 1:500 (Invitrogen, R96025); rabbit polyclonal anti-FLAG (Sigma, F7425; 1:500); mouse monoclonal anti-FLAG (Sigma, F3165; 1:5000); Alexa Fluor-488-conjugated goat anti-rabbit and Alexa-594-conjugated goat anti-mouse antibodies (1:4000; Life Technologies, Invitrogen, A-11008 and A-11005). Images were captured with an Axiophot microscope (Carl Zeiss Inc., ×63, 1.4 numerical aperture Zeiss plan apochromat oil immersion lens) using a cooled CCD camera and at room temperature and Meta View image processing software (Universal Imaging Corp., West Chester, PA). Images were manually adjusted for brightness and contrast with Adobe Photoshop version 7.0.

##### Live Cell Imaging

After 24 h of replating, 18-mm coverslips containing live tsA-201 cells were washed once with Tyrode solution (130 mm NaCl, 2.5 mm KCl, 2 mm CaCl_2_, 2 mm MgCl_2_, 10 mm HEPES, 30 mm glucose, pH 7.4, 319 mosmol/liter), subsequently mounted on a Ludin chamber (Life Imaging Services) and kept in Tyrode solution until further processing. The chamber was placed on an ASI stage of an inverted Zeiss Axiovert 200M epifluorescence microscope (Carl Zeiss). Cells were imaged at room temperature. Different drugs (10 μm oxotremorine M, 50 μm m-3M3FBS (2,4,6-trimethyl-*N*-(meta-3-trifluoromethyl-phenyl)-benzenesulfonamide), 5 μm ionomycin, 20 μm wortmannin, 5 μm rapamycin (Calbiochem, Merck Millipore)) in different combinations (as indicated under “Results”) were diluted to final concentrations in Tyrode solution and either directly added to the imaging chamber or added to a 35-mm culture dish containing the coverslip when preincubation was desired. Twelve-bit grayscale images were recorded at different time intervals using a cooled CCD camera (SPOT, Diagnostic Instruments), Metavue image processing software (Universal Imaging, Corp.), and a ×63, 1.4 numerical aperture Zeiss Plan Apochromat oil immersion lens. For presentation, selected images were linearly adjusted using Adobe Photoshop version 7.0 and CS4. For quantification, areas of interest of equal size were selected in the membrane, cytoplasm, and background of cells, and average pixel intensity was recorded and used to calculate the membrane to cytoplasm ratio for every image. All quantitative data are represented as mean ± S.E. for the indicated number of experiments (*n*), except if stated otherwise. Data were analyzed by paired Student's *t* test, unpaired Student's *t* test, or one-way analysis of variance with Bonferroni post-hoc test using GraphPad Prism version 5.01 (GraphPad Software Inc.). Significance level was set to *p* < 0.05.

##### Electrophysiological Recordings

Whole-cell patch clamp experiments were performed in transiently transfected tsA-201 cells using an Axopatch 200B amplifier (Axon Instruments). Electrodes with a final resistance of 2–4 megaohms were pulled from borosilicate glass capillaries using a P-97 micropipette puller (Sutter Instruments) and subsequently fire-polished (MF-830 microforge, Narishinge). Ca^2+^ currents were measured using the following solutions: pipette solution, 135 mm CsCl, 10 mm Cs-EGTA, 1 mm MgCl_2_, 10 mm HEPES, 4 mm Na_2_-ATP, adjusted to pH 7.3 with CsOH; bath solution, 15 mm CaCl_2_, 150 mm choline chloride, 1 mm MgCl_2_, 10 mm HEPES adjusted to pH 7.3 with CsOH. All voltages were corrected for a junction potential of −9.3 mV. Recordings were digitized (Digidata 1322A digitizer, Axon Instruments) at 50 kHz, low pass-filtered at 5 kHz, and analyzed using pClamp version 10.2 software (Axon instruments). Current-voltage (*I-V*) relationships were obtained by applying a 20-ms square pulse protocol to various test potentials starting from a holding potential of −90 mV. Resulting *I-V* curves were fitted to Equation 1,

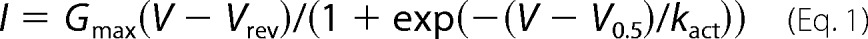
 where *I* is the peak current amplitude, *G*_max_ is the maximum slope conductance, *V* is the test potential, *V*_rev_ is the extrapolated reversal potential, *V*_0.5_ is the half-maximal activation voltage, and *k*_act_ is the activation slope. Channel inactivation was measured using a 300-ms pulse from a holding potential of −90 mV to *V*_max_. For the estimation of the open probability (*P_O_*), the area of the ON-gating current (*Q*_ON_) was integrated and compared with the amplitude of the ionic tail current (*I*_Tail_) at *V*_rev_. Data were analyzed using Clampfit version 10.2 (Axon Instruments) and Sigma Plot 12 (Systat Software Inc.). To assess the effects of diC8-PIP_2_ or the PLC activator m-3M3FBS, cells were depolarized from a holding potential of −90 mV to *V*_max_ for 20 ms at 0.1 Hz. As a control, Ca_V_1.2S- or Ca_V_1.2S_4E_-expressing cells were perfused (flow rate: 250 μl/min) with bath solution only. To test whether diC8-PIP_2_ can stabilize *I*_Ca_ decline, the same protocol was performed in the presence of 100 μm diC8-PIP_2_ in the internal solution. DiC8-PIP_2_ was prepared according to the manufacturer's instructions as a 1 mm stock in intracellular recording solution. Aliquots were kept frozen and diluted before use. To analyze the effects of phosphoinositide depletion on Ca_V_1.2 currents, cells were perfused with 20 μm wortmannin and 50 μm m-3M3FBS. (flow rate: 250 μl/min). All quantitative data are represented as mean ± S.E. Statistical significance was determined by one- or two-way analysis of variance followed by Bonferroni post-hoc test or unpaired Student's *t* test as indicated using GraphPad Prism version 5.01 (GraphPad Software Inc.). Significance level was set to *p* < 0.05.

##### Rosetta Membrane Modeling of the Domain I-II Linker Region and Voltage-sensing Domain II of Ca_V_1.2 Channel

Homology, *de novo*, and full-atom modeling of the voltage-sensing domain (VSD) of native and mutant Ca_V_1.2 channels was performed using the Rosetta membrane method (39–41) and the x-ray structure of the bacterial voltage-gated Na^+^ channel (Na_V_Ab) VSD (42) as a template. Sequence alignment between native Ca_V_1.2 and Na_V_Ab VSDs shown in Fig. 8*A* was generated using the HHpred server (43, 44). The backbone structure of the transmembrane regions of Ca_V_1.2 was built based on Na_V_Ab VSD template. The 19-residue N-terminal region and S1-S2, S2-S3, and S3-S4 loops of Ca_V_1.2 VSD were built *de novo* using the Rosetta cyclic coordinate descent loop modeling method (45) guided by membrane environment-specific energy function (39, 46). 10,000 models were generated for each Ca_V_1.2 channel construct, and the top 10% of models ranked by total score were clustered (47) using root mean square deviation threshold that generates at least 150–200 models in the largest cluster. Models representing centers of the top five clusters and the best 10 models by total score were chosen for visual analysis. The top cluster and all 10 lowest energy models of native Ca_V_1.2 showed very similar conformation of the domain I-II linker region (see Fig. 8). None of the top five clusters and 10 lowest energy models of alanine or glutamate mutants of Ca_V_1.2 showed similar conformations of the domain I-II linker region (see Fig. 8). All structural figures were generated using the UCSF Chimera package (48).

## Results

We (49) and others (50) have recently reported that the I-II linker of LTCC Ca_V_1.3 and Ca_V_1.2 α_1_-subunits is targeted to the plasma membrane when expressed alone as a soluble protein in tsA-201 cells. Because the I-II linker harbors the high affinity interaction site for Ca^2+^ channel β-subunits (51), it is also capable of targeting co-expressed β-subunits to the plasma membrane, even in complex with β_1_-subunit-bound Rab3-interacting molecule (49). Plasma membrane association of the linker is independent of β-subunits. Introduction of mutation W441A, which disrupts β-subunit binding to the linker (31, 52), prevented β-subunit targeting without affecting the plasma membrane localization of the linker (49). This suggested a specific, β-subunit-independent interaction of the I-II linker with the plasma membrane either through a membrane-associated protein or through direct interaction with membrane lipids.

### 

#### 

##### I-II Linkers of All LTCC Isoforms Bind to the Plasma Membrane

**FIGURE 1.**
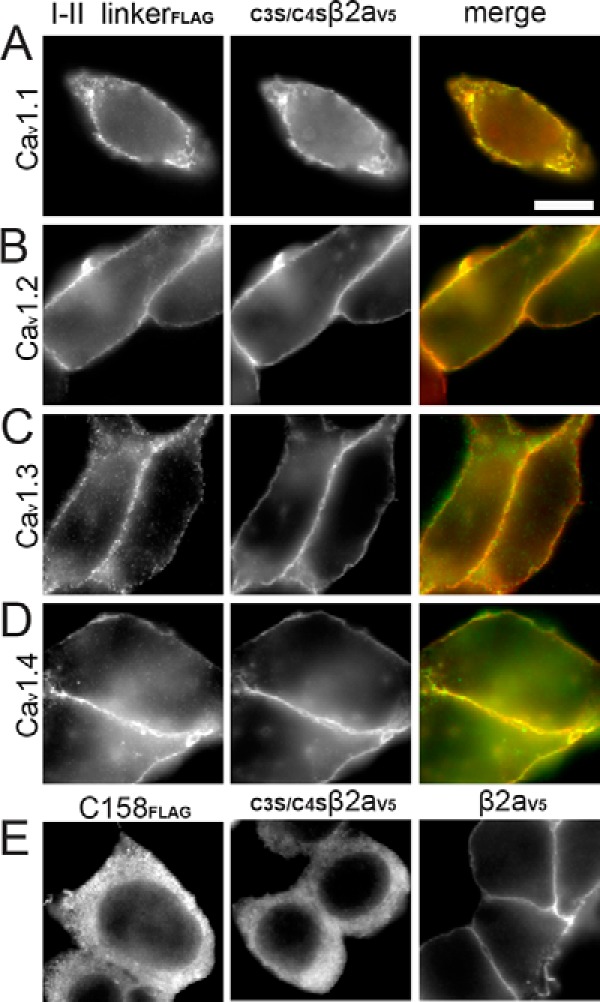
**All LTCC I-II linkers target to the plasma membrane in tsA-201 cells.** Immunofluorescence images of anti-FLAG-labeled LTCC (Ca_V_1.1–Ca_V_1.4) I-II linkers and anti-V5-labeled palmitoylation-deficient β_2a_(_C3S/C4S_β2a_V5_) co-expressed in tsA-201 cells. *A–D*, *left*, anti-FLAG-labeled I-II linkers (I-II linker_FLAG_); *middle*, anti-V5-labeled _C3S/C4S_β_2a_ (_C3S/C4S_β_2aV5_); *right*, merged images (*green*, anti-FLAG-labeled I-II linkers; *red*, anti-V5-labeled _C3S/C4S_β_2a_). *E*, expression of only anti-FLAG-labeled distal 158 amino acids of the Ca_V_1.3 α_1_ C terminus (C158_FLAG_), of anti-V5-labeled _C3S/C4S_β_2aV5_, and of anti-V5-labeled palmitoylated β_2a_ (β2a_V5_), respectively. Representative cells from three independent experiments are shown. *Scale bar*, 10 μm.

To test whether plasma membrane binding is a property of all LTCC α_1_-subunits, we transfected FLAG-labeled I-II linkers of Ca_V_1.1, Ca_V_1.2, Ca_V_1.3, and Ca_V_1.4 α_1_-subunits (Fig. 1, *A–D*, *left*) into tsA-201 cells. Immunoblot analysis (not shown; *n* = 3) confirmed their expression as intact polypeptides. All LTCC I-II linkers were localized at the plasma membrane (Fig. 1, *A–D*). This localization pattern was independent of expression levels and indistinguishably observed in cells with weak and strong expression of the respective linkers (not shown). More than 85% of transfected cells (three independent experiments; 300 cells/experiment analyzed) showed this typical plasma membrane binding. In contrast, a FLAG-labeled control fragment derived from the Ca_V_1.3 C terminus (FLAG-C158) clearly revealed a cytoplasmic distribution (Fig. 1*E*, *first panel*), as did the non-palmitoylated β_2a_ mutant _C3S/C4S_β_2a_ (Fig. 1*E*, *middle*). In contrast, normal palmitoylated β_2a_ revealed the expected plasma membrane targeting (Fig. 1*E*, *last panel*) and thus served as a positive control. Consistent with our previous findings, _C3S/C4S_β_2a_ (*n* = 3; Fig. 1, *A–D*, *middle panel*) and β_3_ (not shown, *n* = 3) (49) also were localized at the plasma membrane after co-expression with one of the four LTCC I-II linkers. This shows that all LTCC I-II linkers support β-subunit plasma membrane targeting.

##### A Polybasic Cluster of Amino Acids Is Essential for I-II Linker Plasma Membrane Binding

**FIGURE 2.**
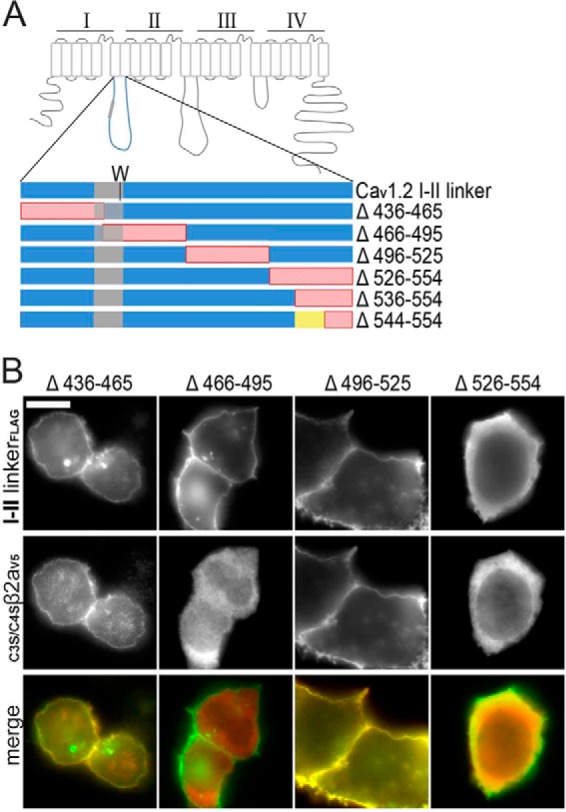
**Plasma membrane localizing signal is present in Ca_V_1.2 I-II linker distal terminus.** *A*, schematic representation of all deletion mutants of the Ca_V_1.2 I-II linker shown in *B*. A tryptophan residue (*W*) important for α_1_-β subunit interaction marks the position of the AID domain (*gray*). *Red*, deletions; *yellow*, amino acids 536–544. *B*, immunofluorescence images of anti-FLAG-labeled deletion mutants of the Ca_V_1.2 I-II linker (*top*), anti-V5-labeled _C3S/C4S_β_2a_ (*middle*), and merged images (*green*, anti-FLAG-labeled I-II linkers; *red*, anti-V5-labeled _C3S/C4S_β_2a_) co-expressed in tsA-201 cells. Representative images from three independent experiments are shown. *Scale bar*, 10 μm.

**FIGURE 3.**
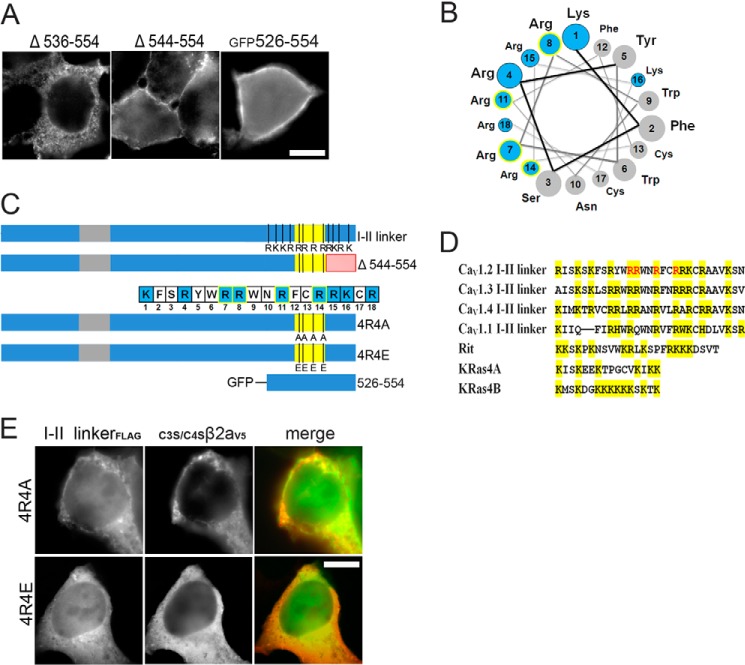
**Positively charged amino acids in region 536–544 are essential for Ca_V_1.2 I-II linker targeting.** *A*, immunofluorescence images of anti- FLAG-labeled deletion mutants as indicated and of _GFP_526–554. Staining of Δ536–554 was always intracellular but with a granular appearance. This was only very rarely seen with Δ526–554, which was typically of uniform distribution (*cf*. Fig. 2*B*). *B*, helical wheel representation of the proposed α-helix with positive charges indicated in *blue*. The 4 arginines important for membrane translocation are *highlighted* in *blue* with a *yellow outline* (*A* and *B*). *C*, schematic representation of positive residues (*R* and *K*) present in 526–554 sequence (Ca_V_1.2 I-II linker). The four important arginines (Arg-537, Arg-538, Arg-541, and Arg-544) in amino acid sequence insert Δ544–554 and mutations (4R4A and 4R4E) in the Ca_V_1.2 I-II linker are indicated. GFP-tagged 526–554 is also shown. *Red*, deletion in 544–554; *yellow*, 536–544; *gray*, α_1_-β subunit interaction site. *D*, putative phosphoinositide-binding domains of all LTCC I-II linkers are compared with published phosphoinositide-binding domains of Rit and K-Ras (22). All basic residues are *marked* in *yellow*, and ones that bind phosphoinositides are *highlighted* in *red. E*, immunofluorescence images of anti-FLAG-labeled charge neutralization linker mutants I-II 4R/4A and I-II 4R/4E (*left*), anti-V5-labeled _C3S/C4S_β_2a_ (*middle*), and *merged images* (*green*, anti-FLAG-labeled I-II linkers; *red*, anti-V5-labeled _C3S/C4S_β_2a_) expressed in tsA-201 cells. Representative images from three independent experiments are shown. *Scale bar*, 10 μm.

To further identify the structural motif within the linker responsible for membrane targeting we expressed triple-FLAG-labeled linker peptides with deletions of equal size (29–30 amino acids) located in different parts of the Ca_V_1.2 linker (Fig. 2*A*). The I-II linker of Ca_V_1.2 was chosen for this analysis because it provided the best signal/noise ratio (not shown). Whereas membrane binding of mutants Δ436–465, Δ466–495, and Δ496–525 remained unaffected by the deletions (Fig. 2*B*), removal of amino acids 526–554 (Fig. 2, *A* and *B*; Δ526–554) abolished plasma membrane binding and resulted in cytoplasmic staining. Accordingly, mutants Δ436–465 and Δ496–525 translocated co-expressed _C3S/C4S_β_2a_ to the plasma membrane (Fig. 2*B*), whereas Δ526–554 did not (Fig. 2*B*). Mutant Δ466–495 failed to target _C3S/C4S_β_2a_ to the plasma membrane (Fig. 2*B*). This was expected, because the β-subunit α_1_-subunit interaction domain (AID) is also removed by this deletion. By creating additional deletion mutants Δ536–554 (no membrane binding; Fig. 3*A*) and Δ544–554 (membrane-bound; Fig. 3*A*) we found that essential structural determinants for membrane targeting must be localized within positions 536–543 (Fig. 2*A*).

Secondary structure analysis of the Ca_V_1.2 I-II linker using PSIPRED (53) (not shown) predicted the region between amino acids 531 and 550 to form a polybasic amphipathic α-helix with 8 of the 9 positive charges (Fig. 3*B*) located on one side of the helix as shown in a helical wheel plot (Fig. 3*B*). This included four arginines (Arg-537, Arg-538, Arg-541, and Arg-544) located within the region 536–544 required for membrane translocation (Fig. 3*C*, *yellow*), all of which are conserved among the I-II linkers of LTCCs (Fig. 3*D*). To test whether these positive charges are essential for plasma membrane binding, we neutralized them by mutations to alanines (mutant I-II-4R4A) or converted them to negatively charged glutamates (mutant I-II-4R4E; Fig. 3*C*). As expected, both mutations prevented the plasma membrane binding of the Ca_V_1.2 I-II linker and of the co-expressed _C3S/C4S_β_2a_ subunits (Fig. 3*E*).

**FIGURE 4.**
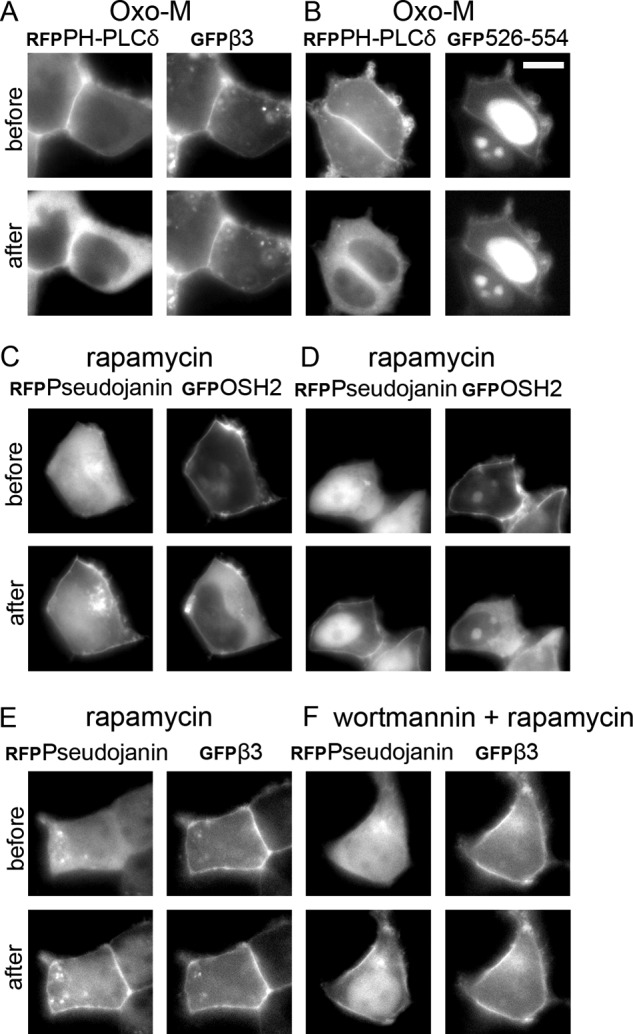
**Plasma membrane interaction of Ca_V_1.2 I-II linker is not influenced by muscarinic receptor activation or rapamycin induced PIP or PIP_2_ depletion.** *A* and *B*, mRFP-labeled PH-PLCδ and GFP-labeled β_3_ with untagged Ca_V_1.2 I-II linker or mRFP-labeled PH-PLCδ and GFP-labeled 526–554 were co-expressed with untagged muscarinic M1 receptor in tsA-201 cells and subsequently treated with the M1 receptor agonist Oxo-M (10 μm). Fluorescence was visualized using live cell imaging, and images before (*t* = 0 s) and after (*t* = 5 s) the drug application were recorded. *A*, *left*, mRFP-labeled PH-PLCδ; *right*, GFP-labeled β_3_ in the same cells co-expressed with untagged Ca_V_1.2 I-II linker and mRFP-labeled PH-PLCδ. *B*, *left*, mRFP-labeled PH-PLCδ; *right*, GFP-labeled peptide 526–554 in the same cells co-expressed with mRFP-labeled PH-PLCδ. Representative cells from three independent experiments are shown. *C–F*, cells co-transfected with Lyn_11_-FRB, RFP-pseudojanin, and GFP-PH-Osh2x2 or Ca_V_1.2 I-II linker together with β_3_-GFP. Fluorescence was visualized before (*t* = 0 s) and after drug (*t* = 60 s) application. *C* and *D*, RFP-labeled pseudojanin (*left*) with GFP-labeled PH-Osh2x2 (*right*) as control before and after rapamycin treatment. Representative cells from eight independent experiments are shown. *E*, *left*, RFP-labeled pseudojanin; *right*, GFP-labeled β_3_, before and after rapamycin application. Representative cells from three independent experiments are shown. *F*, *left*, RFP-labeled pseudojanin; *right*, GFP-labeled β_3_ preincubated with wortmannin (*before*) and after the addition of rapamycin (*after*). Representative cells from three independent experiments are shown. *Scale bar*, 10 μm.

**FIGURE 5.**
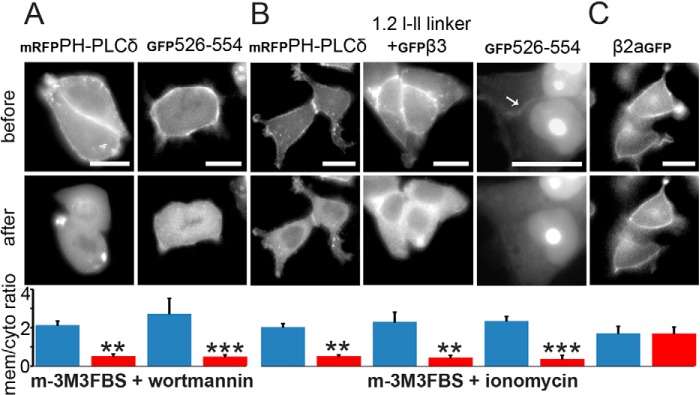
**Reversal of I-II linker-induced plasma membrane targeting due to phosphoinositide depletion accompanied by increased intracellular Ca^2+^ levels.** *A*, mRFP-labeled PH-PLCδ and GFP-labeled 526–554 were co-expressed with untagged muscarinic M1 receptor in tsA-201 cells treated with the PLC activator m-3M3FBS (50 μm) together with wortmannin (20 μm); *left*, mRFP-labeled PH domain of PLCδ; *right*, GFP-labeled 526–554. Representative cells from three independent experiments are shown. *B* and *C*, different constructs were expressed in tsA-201 cells and subsequently treated with the PLC activator m-3M3FBS (50 μm) and additional ionomycin (5 μm). *B*, *left*, mRFP-labeled PH-PLCδ; *middle*, GFP-labeled β_3_-subunit co-expressed with Ca_V_1.2 I-II linker; *right*, GFP-labeled peptide 526–554. The membrane-localized staining reversed by treatment is indicated by an *arrow*. Note that in the majority of cells, _GFP_526–554 also showed strong nuclear targeting (for an explanation, see “Results”), which was absent in dividing cells without distinct nuclei (Figs. 3*A* and 5*A*). *C*, GFP-labeled β_2a_ alone. To quantify the relocation from the membrane, pixel intensity from three representative areas of interest within the membrane of each cell were background-corrected, and the membrane/cytoplasm ratio was calculated from the means obtained before (*blue*) and after (*red*) PLC activation. Means ± S.E. (*error bars*) are shown for the indicated number of cells. Paired Student's *t* test was used. **, *p* = 0.0049 (PH-PLCδ, m-3M3FBS + wortmannin, *n* = 3); ***, *p* = 0.00016 (peptide 526–554, m-3M3FBS + wortmannin, *n* = 3); **, *p* = 0.00427 (PH-PLCδ, m-3M3FBS + ionomycin, *n* = 15); **, *p* = 0.01087 (β_3_-subunit + Ca_V_1.2 I-II linker, m-3M3FBS + ionomycin, *n* = 5); ***, *p* = 0.0010 (peptide 526–554, m-3M3FBS + ionomycin, *n* = 3); *p* = 0.81799 (β_2a_, m-3M3FBS + ionomycin, *n* = 6). *Scale bar*, 10 μm.

To assess whether this polybasic motif is sufficient to induce plasma membrane binding when attached to an otherwise cytoplasmic protein, we fused residues 526–554 to the C terminus of GFP (_GFP_526–554; Fig. 3, *A* and *C*). This construct localized to the plasma membrane (Fig. 3*A*). It also accumulated in the nucleus of the vast majority of transfected cells. Notably, a distinct feature of lipid-interacting polybasic membrane targeting motifs is their similarity to nuclear localization sequences (22), a property that may also account for the nuclear targeting of _GFP_526–554. However, nuclear staining did not obscure plasma membrane binding (see also Figs. 4*A* and 5*B*), and this was also confirmed in dividing cells in which nuclear staining was completely absent (Fig. 3*A*, _GFP_526–554, *right panel*; see also Fig. 5*A*). Taken together, these findings clearly demonstrate that positive charges at the C-terminal end of the Ca_V_1.2 I-II linker form a plasma membrane binding motif sufficient for translocating the cytoplasmic I-II linker and fused GFP to the plasma membrane.

##### Involvement of Membrane Phosphoinositides in Plasma Membrane Targeting

The polybasic motif at the distal end of the I-II linker closely resembles clusters of positive charges found in other proteins, such as Rit and K-Ras (22) (Fig. 3*D*), and polybasic domains in other ion channels mediating protein-lipid interactions at the plasma membrane (for a review, see Ref. 13). We therefore hypothesized that this cluster of positive charges could serve a similar function. This notion was further supported by current folding models of Ca^2+^ channel α_1_-subunits, which position these charges near the cytoplasmic end of the transmembrane helix IIS1 (42), which, in turn, places them in close proximity to the anionic lipids of the inner leaflet of the plasma membrane.

Many modulatory lipid interactions of ion channels involve polyphosphoinositides (13). Ca_V_1 and Ca_V_2 VGCCs are modulated by metabotropic receptors through activation of PLC, also independently of direct G-protein modulation (16). Activation of M1 receptors induces a partial inhibition of Ca^2+^ inward currents mostly by membrane depletion of PIP_2_ in tsA-201 cells (16). This effect is mediated via activation of PLCβ (54). If binding of LTCC I-II linkers is dynamically regulated by interaction with PLC-metabolized polyphosphoinositides (primarily PIP_2_), then it should be reversed in a PLC-sensitive manner.

We therefore co-transfected tsA-201 cells with M1 receptors and examined effects on membrane binding of our constructs in response to receptor activation with oxotremorine M (Oxo-M) using live cell imaging (Fig. 4, *A* and *B*). The PIP_2_-specific mRFP-tagged pleckstrin homology domain of PLCδ (PH-PLCδ) served as positive control for monitoring PIP_2_ breakdown (55). Oxo-M induced the relocation of PH-PLCδ but did not reverse the plasma membrane association of I-II linker-bound β_3_-GFP (*n* = 3 independent experiments; Fig. 4*A*) or of _GFP_526–554 (*n* = 3; Fig. 4*B*). Translocation of membrane-bound β_3_-subunits was also absent in cells responding to co-transfected PH-PLCδ (*n* = 3), excluding a potential lack of Oxo-M action as a possible explanation. Even during prolonged observation times (10 min; not shown), no translocation was observed. As reported previously (55), Oxo-M-induced PIP_2_ depletion was transient, resulting in a relocation of the PIP and PIP_2_ biosensor GFP-PH-Osh2x2 (37) (5 of 21 cells) and PH-PLCδ (3 of 21 cells; not shown) within 2 min despite the continuous presence of Oxo-M. This is compatible with incomplete PIP/PIP_2_ depletion in mammalian cells by Oxo-M (55) under these experimental conditions.

Previous studies have shown that plasma membrane targeting of cytoplasmic proteins through cationic motifs resembling our polybasic sequence may also involve negatively charged lipids other than PIP_2_, in particular PIP_3_, PIP (22), or phosphatidylserine (21). We therefore used a rapamycin-inducible system (37) causing PIP and PIP_2_ depletion. Briefly, RFP-tagged pseudojanin (PJ), an enzymatic chimera that converts PIP_2_ to PIP and dephosphorylates PIP, was translocated from the cytosol to the plasma membrane upon rapamycin-induced Lyn_11_-FRB interaction. Translocation of PJ to the plasma membrane started 15.6 ± 3.12 s after rapamycin application and was completed after 30.4 ± 3.61 s in the control experiments (Fig. 4, *C* and *D*). This was followed by membrane dissociation of co-transfected GFP-PH-Osh2x2 (starting 29.6 ± 4.03 s after drug administration, completed after 57.1 ± 3.28 s; mean ± S.E., *n* = 24). Notably, in 13 of 24 cells, some residual membrane staining of GFP-PH-Osh2x2 still remained (Fig. 4*C*). When we co-transfected the rapamycin-inducible system and the I-II linker together with β_3_-GFP, PJ translocation started at 13.1 ± 1.43 s and was completed within 25.3 ± 1.07 s (mean ± S.E., *n* = 16) after rapamycin application. However, even after a prolonged recording time (20 min), we never observed a I-II linker-mediated translocation of β_3_-GFP to the cytoplasm (*n* = 16 cells; three independent experiments; Fig. 4*E*). We also combined the rapamycin-inducible system with wortmannin treatment. Preincubation with 20 μm wortmannin for 45–90 min before rapamycin application did not affect targeting of controls (PJ translocation started only upon rapamycin application after 20 ± 3.33 s, completion after 37.0 ± 5.17 s, *n* = 10) or linker-bound β_3_-GFP (*n* = 10, three independent experiments; Fig. 4*F*).

Earlier studies have shown very strong plasma membrane binding of similar polybasic targeting motifs of small GTPases, such as Rit or K-Ras (21, 22) (Fig. 3*D*) or the plasma membrane targeting domain of RasGRP1, a Ras-specific exchange factor (56). Their dissociation requires, in addition to phosphoinositide hydrolysis, either depletion of PI3K products (22, 56) or prolonged elevation of intracellular Ca^2+^ (21). Because neither the rapamycin-inducible system nor the Oxo-M response appears to induce complete phosphoinositide depletion and induces no or, in the case of Oxo-M, only a transient intracellular Ca^2+^ signal (55), we employed the PLC activator m-3M3FBS together with wortmannin to induce PIP, PIP_2_, and PIP_3_ depletion and inhibit PI3K. m-3M3FBS activates PLCβ, -γ, and -δ isoforms and also induces a prolonged intracellular Ca^2+^ release (55, 57). Application of 50 μm m-3M3FBS alone was not sufficient to induce translocation within our observation period. However, co-application of m-3M3FBS together with wortmannin caused a delayed translocation of PH-PLCδ (*n* = 4; Fig. 5*A*, *left*) as well as of _GFP_526–554 (*n* = 11 independent experiments; Fig. 5*A*, *right*) from the plasma membrane to the cytosol starting within a time frame of 20–30 min and completion in the following 7–10 min. This suggested a combined effect of intracellular Ca^2+^ (which acts as a PLC co-activator (55)) and lipid hydrolysis for slow translocation. Accordingly, the addition of 5 μm ionomycin together with m-3M3FBS (58) induced an even faster relocation of PH-PLCδ to the cytoplasm (Fig. 5*B*, *left*). Translocation started 1.9 ± 0.2 min (mean ± S.D., *n* = 15) after m-3M3FBS/ionomycin stimulation and was complete within 45–55 s. When we treated cells co-transfected with the I-II linker together with β_3_-subunits, PLC activation reversed the linker-mediated β_3_-GFP association with the plasma membrane (Fig. 5*B*, *middle*). Although cell to cell differences for the translocation time course after adding m-3M3FBS/ionomycin were observed, the translocation of β_3_-GFP and PH-PLCδ always occurred in parallel (*n* = 5 independent experiments; not shown). The GFP-tagged cationic peptide (_GFP_526–554) also redistributed to the cytoplasm under these experimental conditions (*n* = 3; Fig. 5*B*, *right*). In contrast, no translocation was observed in control experiments with β_2aGFP_ subunits, which are anchored to the membrane through palmitoylation, known to be insensitive to polyphosphoinositide breakdown (*n* = 6; Fig. 5*C*). Taken together, our data demonstrate that polyphosphoinositide breakdown as well as increased intracellular Ca^2+^ levels are required for unbinding of the distal portion of the Ca_V_1.2 I-II linker from the plasma membrane.

##### Role of the Polybasic Motif for Ca_V_1.2 Channel Function and Modulation

Due to the close proximity of the polybasic motif to the membrane and its lipid-dependent interaction with the plasma membrane, our data strongly suggest that this interaction also occurs in the pore-forming α_1_-subunit of intact Ca_V_1.2 channel complexes. We therefore introduced the mutations found to prevent membrane binding into the I-II linker constructs I-II-4R/4A and I-II-4R/4E (Fig. 3*D*) into the corresponding positions of the intact Ca_V_1.2 α_1_-subunits and expressed these mutants (together with β_3_ and α_2_δ_1_) in tsA-201 cells. Mutation-induced changes of Ca^2+^ inward current (*I*_Ca_) properties were then analyzed using whole-cell patch clamp experiments with Ca^2+^ as charge carrier. Ca_V_1.2 α_1_-subunit channels undergo partial proteolytic processing, giving rise to a C-terminally long (Ca_V_1.2L) and short (Ca_V_1.2S) variants. Both variants exist in the heart and brain and exhibit different current properties (59, 60). In particular, Ca_V_1.2S exhibits a higher open probability (59, 60). We therefore tested the effects of the I-II linker mutations in both variants (long, mutants Ca_V_1.2L_4A_ and Ca_V_1.2L_4E_; short, mutants Ca_V_1.2S_4A_ and Ca_V_1.2S_4E_).

**FIGURE 6.**
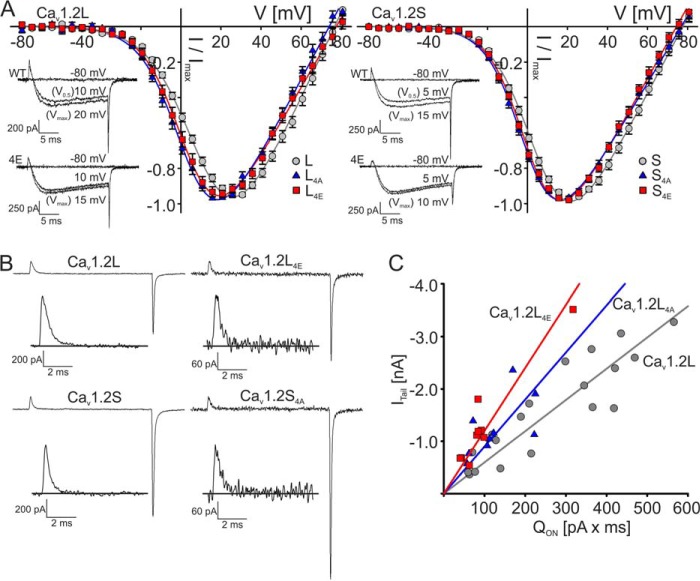
**Biophysical properties of wild-type and mutant Ca_V_1.2L and Ca_V_1.2S.** Parameters and statistics are given in Table 1. *A*, normalized current-voltage (*I-V*) relationships of Ca_V_1.2L (*gray circles*), Ca_V_1.2L_4A_ (*blue triangles*), and Ca_V_1.2L_4E_ (*red squares*) (*left*) and Ca_V_1.2S (*gray circles*), Ca_V_1.2S_4A_ (*blue triangles*), and Ca_V_1.2S_4E_ (*red squares*) (*right*) measured by a 20-ms depolarization step to various test potentials using 15 mm Ca^2+^ as a charge carrier. Data points are represent means ± S.E. (*error bars*). *Insets*, representative traces for Ca_v_1.2L (*top left*), Cav1.2L_4E_ (*bottom left*), Cav1.2S (*top right*), and Ca_v_1.2S_4A_ (*bottom right*) for the indicated voltages. Note that at test potentials close to the *V*_0.5_ of wild-type channels, the mutant channels exhibit current amplitudes closer to *V*_max_ due to a shift of the *I-V* relationship toward more negative voltages. *B*, representative current traces of Ca_V_1.2L (*top left*) *versus* Ca_V_1.2L_4E_ (*top right*) and Ca_V_1.2S (*bottom left*) *versus* Ca_V_1.2S_4A_ (*bottom right*) obtained by depolarizing the cell from −90 mV to *V*_rev_. *Insets*, *enlarged Q*_ON_ of the *traces. C*, correlation of *Q*_ON_ (area) with maximum tail current amplitude (*I*_Tail_) measured at *V*_rev_ for wild-type and mutant Ca_v_1.2S. The *color code* is as in *A*. The following slopes were obtained by linear regression analysis: −12.2 ± 1.13 for Ca_v_1.2S (*R*^2^ = 0.64), −17.2 ± 1.14 for Ca_v_1.2S_4A_ (*R*^2^ = 0.82), −27.7 ± 1.73 for Ca_v_1.2S_4E_ (*R*^2^ = 0.70). Slopes were significantly different for each data set (*p* < 0.0001; F-test: *F* (DFn, DFd) = 15.6 (2, 34)) as well as for Ca_v_1.2L and corresponding mutants (*p* < 0.0001; not shown).

All four mutant constructs conducted inward Ca^2+^ currents. Current-voltage relationships revealed a significant 6–10 mV shift of *V*_0.5_ in the hyperpolarizing direction for both mutants (Table 1 and Fig. 6*A*). This was due to a significant increase in the slope without changes in activation threshold (Table 1), suggesting more efficient coupling of pore opening to membrane depolarization. The mutations had little effect on the kinetics of *I*_Ca_ inactivation during 300-ms depolarizing pulses to the voltage corresponding to the peak of the *I-V* relationship (Table 2). The apparent reversal potential was also unchanged (Table 1). Differences between the mutants and wild-type channels were observed when we studied the relationship between the size of ON-gating charges (as a measure of active channels on the cell surface; enlarged ON-gating currents are shown in the *insets* of Fig. 6*B*) and the size of ionic tail currents (Fig. 6*B*). As shown by us and others previously, this ratio provides an estimate for the channel's open probability (61–63). The two wild-type constructs served as an internal control because a higher open probability was reported earlier for Ca_V_1.2S (59, 61). This is evident as a statistically significant difference of the *I*_Tail_/*Q*_ON_ ratio in our experiments (Fig. 6*B*; see Table 1 for statistics). This is also evident from the steeper slopes of the regression lines of plots of *I*_Tail_ *versus Q*_ON_ (Fig. 6*C*). In Ca_V_1.2L, the 4R/4A mutation caused a 64% increase in the *I*_Tail_/*Q*_ON_ ratio. A similar increase was also observed in the Ca_V_1.2S mutants despite higher basal open probability. An even larger effect was seen for the Ca_V_1.2L_4E_ and Cav1.2S_4E_ mutants, which more than doubled the open probability. We also found that all four arginine mutant constructs significantly reduced *Q*_ON_ in both Ca_V_1.2L and Ca_V_1.2S, with the reduction again more pronounced for the two 4R/4E mutations. Our data demonstrate that positive charges located in proximity to the transmembrane segment IIS1 within the I-II linker are important determinants of normal Ca_V_1.2 Ca^2+^ channel function independent of the length of their C-terminal tails and basal open probabilities. The negative shift in *V*_0.5_ and higher open probability both indicate a tighter coupling between the voltage sensor and the pore. Based on our membrane targeting analysis, we propose that we have identified a site that is involved in the membrane lipid-dependent stabilization of channel function as well as the regulation of the expression of functional channels at the plasma membrane.

**TABLE 1:** **Biophysical properties of wild type and mutant Ca_V1.2L_ and Ca_V1.2S_** Parameters are indicated as means ± S.E. for a given number of experiments (*n*). Statistical significances are indicated for comparisons of Ca_V_1.2L *versus* Ca_V_1.2L_4A_ and Ca_V_1.2L_4E_ (*a–aaa*), Ca_V_1.2S *versus* Ca_V_1.2S_4A_ and Ca_V_1.2S_4E_ (*b–bbb*) (one-way analysis of variance, Ca_V_1.2L: *V*_0.5_ *p* < 0.0001, *k*_act_ *p* < 0.0069, *I*_Tail_/*Q*_ON_ *p* < 0.0001; Ca_V_1.2S: *V*_0.5_ *p* < 0.0016, *k*_act_ *p* < 0.0216, *P_o_ p* < 0.0001; with Bonferroni post-hoc test as indicated in the table), and Ca_V_1.2L *versus* Ca_V_1.2S (*c–ccc*) (unpaired Student's *t* test). Significance was defined as *p* < 0.05 (*a*, *b*, and *c*), *p* < 0.01 (*aa*, *bb*, and *cc*), and *p* < 0.001 (*aaa*, *bbb*, and *ccc*). For reliable calculation of *Q*_ON_, we omitted data from recordings in which *Q*_ON_ was not larger than 4-fold the signal of noise (5 of a total of 79 recordings).

Ca_V_1.2 construct	*V*_0.5_	Activation threshold	*k*_act_	*V*_rev_	*n*	*I*_Tail/*Q*^ON^_	*Q*_ON_ mean (min; max)	*n*
	*mV*	*mV*	*mV*	*mV*		*ms*^−*1*^	pA*ms	
Ca_v_1.2L	13.96 ± 1.64	−31.58 ± 1.28	11.24 ± 0.45	77.08 ± 1.19	28	6.54 ± 0.47	260.5 (59.8; 565.4)	19
Ca_v_1.2L_4A_	3.47 ± 1.53*^aaa^*	−33.40 ± 0.80	9.62 ± 0.24*^a^*	73.56 ± 1.28	12	10.73 ± 1.36*^aa^*	127.5 (57.4; 224.7)*^a^*	9
Ca_v_1.2L_4E_	4.07 ± 1.90*^aa^*	−30.81 ± 1.59	9.30 ± 0.34*^a^*	77.72 ± 2.16	11	14.00 ± 1.27*^aa^*	100.4 (40.1; 317.8)*^aa^*	9
Ca_v_1.2S	7.03 ± 1.41*^cc^*	−32.09 ± 0.81	10.06 ± 0.38	77.20 ± 1.28	22	13.35 ± 1.46*^ccc^*	266.2 (101.2; 674.7)	12
Ca_v_1.2S_4A_	0.77 ± 1.22*^bb^*	−32.63 ± 0.72	8.95 ± 0.28*^b^*	76.69 ± 1.18	22	22.90 ± 2.21*^bb^*	136.1 (46.4; 495.7)*^b^*	14
Ca_v_1.2S_4E_	1.31 ± 1.28*^bb^*	−31.92 ± 0.73	8.85 ± 0.34*^b^*	74.46 ± 1.17	17	31.42 ± 2.07*^bbb^*	76.4 (40.0; 172.0)*^bbb^*	11

**TABLE 2:** **Inactivation properties of wild-type and mutant Ca_V1.2L_ and Ca_V1.2S_** Cells were depolarized for 300 ms from a holding potential of −90 mV to *V*_max_. *R* values represent the remaining fraction of *I*_Ca_ (15 mm) after 50, 100, or 250 ms. Parameters are indicated as means ± S.E. for a given number of experiments (*n*). Statistical significances are indicated for comparisons of Ca_V_1.2L *versus* Ca_V_1.2L_4A_ and Ca_V_1.2L_4E_ (*a*) (one-way analysis of variance with Bonferroni post-hoc test as indicated in the table) and Ca_V_1.2L *versus* Ca_V_1.2S (*b* and *bb*, unpaired Student's *t* test). Significance was defined as *p* < 0.05 (*a* and *b*) and *p* < 0.01 (*bb*).

Ca_V_1.2 construct	R50	R100	R250	*n*
	**%**	**%**	**%**	
Ca_v_1.2L	65.01 ± 2.51	49.39 ± 2.61	32.54 ± 2.02	7
Ca_v_1.2L_4A_	60.77 ± 2.05	46.83 ± 2.23	33.16 ± 2.34	7
Ca_v_1.2L_4E_	56.96 ± 1.67*^a^*	42.60 ± 2.43	29.83 ± 1.64	7
Ca_v_1.2S	52.86 ± 2.37*^bb^*	37.09 ± 2.02*^bb^*	24.72 ± 2.02*^b^*	13
Ca_v_1.2S_4A_	50.63 ± 2.57	36.32 ± 2.31	26.48 ± 2.18	13
Ca_v_1.2S_4E_	52.28 ± 2.46	38.55 ± 2.36	27.22 ± 2.14	13

**FIGURE 7.**
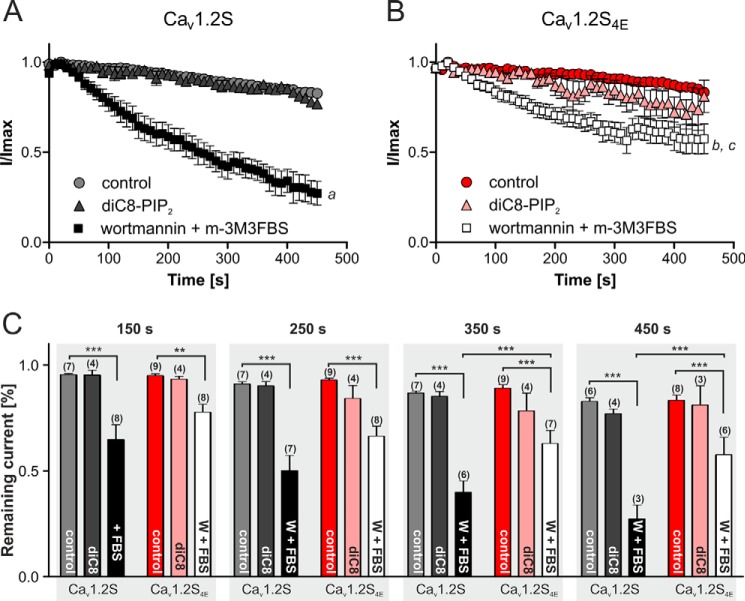
**Modulation of Ca_v_1.2S and Ca_v_1.2S_4E_ currents by wortmannin/m-3M3FBS or diC8-PIP_2_ treatment.** *A*, Ca_v_1.2S, perfused with bath solution (control; *gray circles*; data are shown for seven cells; due to different recording duration, means ± S.E. reflect *n* = 7 until 410 s, *n* = 6 until 450 s), 100 μm intracellular diC8-PIP2 (*gray triangles*; means ± S.E., *n* = 4), or extracellular 20 μm wortmannin and 50 μm m-3M3FBS (*black squares*; means ± S.E. for *n* = 8 until 280 s, *n* = 3 until 450 s). *B*, Ca_v_1.2S_4E_, control (*red circles*; *n* = 9 cells until 440 s, *n* = 8 cells until 450 s), 100 μm intracellular diC8 (*red triangles*; *n* = 4 until 420 s, *n* = 3 until 450 s), or extracellular 20 μm wortmannin and 50 μm m-3M3FBS (*white squares*; *n* = 8 until 320 s, *n* = 6 until 450 s). Statistical analysis was performed using two-way analysis of variance of all data sets followed by Bonferroni post hoc test. Wortmannin/m-3M3FBS treatment significantly enhanced current decrease over time for both constructs (*a*, *p* < 0.01 after 100 s and *p* < 0.001 after 120 s for Ca_v_1.2S; *b*, *p* < 0.05 after 140 s and *p* < 0.001 after 170 s for Ca_v_1.2S_4E_). The wortmannin/m-3M3FBS response was significantly attenuated in Ca_v_1.2S_4E_ *versus* wild type (*c*, *p* < 0.05 after 260 s, *p* < 0.01 or *p* < 0.001 after 330 s). DiC8-PIP2 treatment had no effect and was also not different between wild type and mutant Ca_v_1.2. Because current underwent some minor initial run-up, time 0 was set when the currents reached a stable maximum (1.5–2 min after start of perfusion) to which current amplitudes were normalized. *C*, percentage of remaining current of Ca_v_1.2S and Ca_v_1.2S_4E_ in control-, diC8-, or wortmannin/m-3M3FBS (*W/FBS*)-treated cells after different time points. Data are shown as mean ± S.E. (*error bars*). Statistical significance at the indicated time points was taken from the statistical analysis described above. *, *p* < 0.05; **, *p* < 0.01; ***, *p* < 0.001.

We also tested whether the arginine mutations in Ca_V_1.2S_4E_ (*versus* Ca_V_1.2S as wild-type control) affect the time-dependent decline of whole-cell *I*_Ca_ and the modulation by added PIP_2_ (by intracellular application of a 100 μm concentration of the water-soluble PIP_2_ analogue diC8-PIP_2_, (15, 64)) or phosphoinositide hydrolysis induced by wortmannin (20 μm) plus m-3M3FBS (50 μm) as in live cell imaging experiments (Fig. 5*A*). Perfusion of cells with control solution induced a slow decrease in activity with time (“run-down”), as expected for Ca_v_1.2 channels (65). *I*_Ca_ decline during perfusion with control solution was similar in wild-type and the mutant channel and was also not affected by intracellular application of diC8-PIP_2_ (Fig. 7). However, extracellular perfusion with wortmannin/m-3M3FBS significantly inhibited *I*_Ca_ of wild-type and Ca_v_1.2_4E_ channels, but this current inhibition was significantly attenuated in the mutant channel (Fig. 7). This indicates that membrane association of the distal I-II linker through its positive charges not only stabilizes a more reluctant channel state (Fig. 6) but also weakens inhibition of channel activity by phosphoinositide depletion.

##### Structural Modeling of the Domain I-II Linker Region of Ca_V_1.2 Channel

**FIGURE 8.**
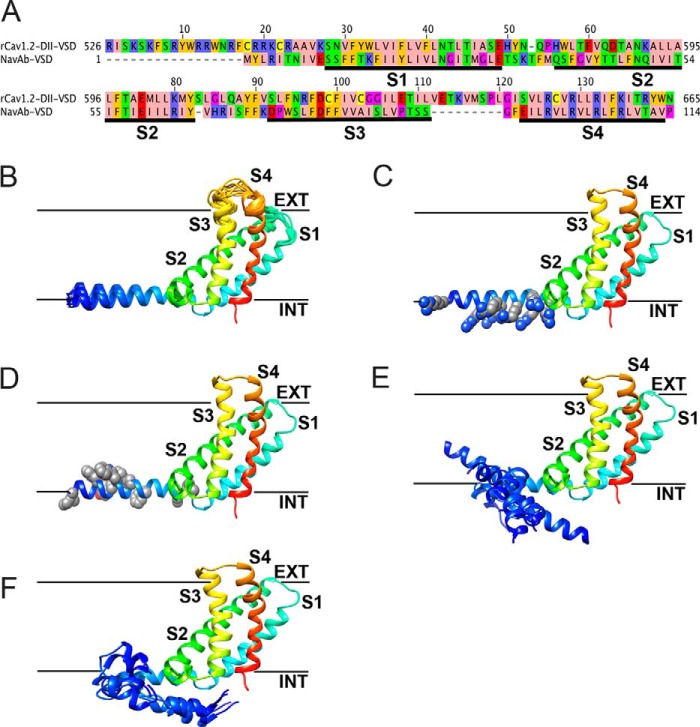
**Structural models of the domain I-II linker region of native Ca_V_1.2 channel.** *A*, sequence alignment between the native Ca_V_1.2 I-II linker region and the domain II voltage-sensing domain (*rCa_V_1.2-DII-VSD*) and *Na_V_Ab* voltage-sensing domain (*Na_V_Ab-VSD*). Transmembrane segments S1–S4 are *underlined* by *black bars* and *labeled*. Amino acids were colored using the Zappo color scheme in Jalview. *B–D*, transmembrane view of the *ribbon representation* of the top cluster models of the VSD of Ca_V_1.2 with the 10 lowest energy Rosetta models superimposed in *B* and *space-filling representations* of arginine side chains in the domain I-II linker helix in *C* and of large hydrophobic side chains in the I-II linker helix in *D. E* and *F*, *transmembrane view* of *ribbon representation* of the top five clusters and 10 lowest energy Rosetta models of the Ca_V_1.2 VSD of alanine mutants superimposed in *E* and of glutamate mutants superimposed in *F*. Models are *colored* in a *rainbow scheme* from *blue* (N-terminal region before S1 segment) to *red* (S4 segment). Transmembrane segments S1–S4 are labeled accordingly. *Black bars*, approximate location of the extracellular and intracellular edges of the hydrophobic layer of the membrane.

To further explore the conformation of this distal domain I-II linker region of the Ca**_V_**1.2 channel (corresponding to residues 536–554 containing the polybasic cluster), its interaction with the inner leaflet of the membrane bilayer, and potential changes introduced by charge neutralizations, we applied the Rosetta membrane method (39–41) and the x-ray structure of the voltage-sensing domain of a recently crystallized bacterial voltage-gated Na^+^ channel, Na_V_Ab (Fig. 8*A*) as described under “Experimental Procedures.” The best models of the native Ca_V_1.2 channel revealed convergence between the top cluster model (the most frequently sampled conformation) and the 10 lowest energy models (Fig. 8*B*). In those models, the distal I-II linker forms a straight helix in which arginine residues are oriented away from the hydrophobic layer of the membrane (*lines* in Fig. 8) and are therefore in a favorable position for possible interactions with lipid phosphate groups (Fig. 8*C*). Large hydrophobic residues in the domain I-II linker region are oriented toward the hydrophobic layer of the membrane, which is in good agreement with their favorable environment based on analysis of available membrane protein structures (Fig. 8*D*). The best models of the domain I-II linker arginines mutated to alanines or glutamates are shown in Fig. 8, *E* and *F*. Unlike in wild-type models, there is no convergence among any of the top cluster models and the best scoring models. Our model suggests that native arginines in the distal I-II linker strongly favor straight helix conformation, positioning it entirely at the interface between the hydrocarbon core and lipid head groups.

Based on Rosetta predictions, the alanine mutations clearly disturb this conformation, making the helical region structurally unstable. We therefore suggest that alanine and glutamate mutations in the domain I-II linker not only affect its plasma membrane binding but also disturb secondary structure, leading to altered positioning of the domain I-II linker helix near the membrane-spanning helices of repeat II.

## Discussion

We describe the identification of a polybasic motif within the I-II linker of the pore-forming α_1_-subunit of voltage-gated Ca_V_1.2 L-type Ca^2+^ channels with biochemical features that allow its attachment to the inner leaflet of the plasma membrane via interaction with negatively charged phospholipids. First, we demonstrate that the I-II linkers of all four LTCC isoforms bind to the plasma membrane in living cells (Fig. 1). This enables this polypeptide to translocate large complexes of cytoplasmic proteins to the plasma membrane (different β-subunits, Rab3-interacting molecule in complex with β-subunits (49)). Second, this interaction is reversible upon pharmacological activation of PLC together with either Ca^2+^ as an additional PLC activator (66) or with wortmannin (Fig. 5), which prevents resynthesis of polyphosphoinositides (22). Under both activating conditions, the time course of membrane unbinding of the I-II linker closely follows the time course of unbinding of the PIP_2_-selective sensor PH-PLCδ. No such translocation was found for β_2a_-subunits, which are membrane-anchored through their palmitoic acid side chain and thus insensitive to PLCs. Together, these findings provide strong experimental evidence that binding of the I-II linker through this polybasic motif to the plasma membrane also involves interaction with polyphosphoinositides. Third, the primary structure of our lipid-binding motif has the characteristics of previously reported polybasic motifs implicated in polyphosphoinositide-interactions of other ion channels or of small GTPases, such as Rit and K-Ras (Fig. 3*C*) (22). The polybasic motifs of these proteins are sufficient for their plasma membrane targeting. Similar to our motif, the removal of only a few residues in Rit was sufficient to completely prevent translocation (3 in Rit, 4 in the I-II linker) (22). Moreover, positively charged clusters required for interaction with membrane lipids have recently also been identified in the auxiliary β_2e_ subunit of VGCC (67).

Using *de novo* molecular modeling, we predict this membrane binding motif to form a straight α-helix positioned entirely at the interface between the hydrocarbon core and lipid headgroups, with the positive charges facing the lipid phosphates. Moreover, according to the model, the four arginines not only support plasma membrane binding of the I-II linker but are also necessary to stabilize this secondary structure in the context of the membrane environment. This stabilization may also explain the functional changes we observed. We demonstrate that the four basic residues are by themselves determinants of Ca_V_1.2 Ca^2+^ channel function. In two different Ca_V_1.2 α_1_-subunit variants with distinct intrinsic open probabilities (61), neutralization of these charges enhanced Ca_V_1.2-mediated inward currents resulting from increased tail Ca^2+^ currents relative to total ON-gating charge. In addition, these mutations also shifted half-maximal activation to more negative potentials by increasing the slope of the current-voltage relationship. Both observations indicate that charge neutralization (or conversion) enhances the coupling between voltage sensing and pore opening. This is in accordance with the previous finding that the I-II linker encodes self-reliant molecular motifs for channel activation independent of β-subunits (68, 69). Our data show that residues remote from the β-subunit interaction site in the I-II linker are required for stabilization of normal Ca_V_1.2 channel function. Although only crystallization studies such as those recently described for Kir2.2 and GIRK2 K^+^ channels (23, 24) can ultimately confirm the direct interaction of channel domains with membrane lipids, our data provide strong evidence that the polybasic motif described here constitutively stabilizes channel function through membrane lipid binding. In addition, we also found that charge neutralization and conversion also reduced *Q*_ON_, revealing a reduction of functional channels at the plasma membrane. This strongly indicates that the interaction of this basic motif with negatively charged membrane lipids is also important for regulating the expression of functional Ca_v_1.2 channels at the membrane surface.

Previous studies have identified other potential PIP_2_ interaction sites within VGCC α_1_-subunits. Mutation of Ile-1520 in segment IIIS6 of the Ca_V_2.1 α_1_-subunit reduced current run-down attributed to PIP_2_ depletion (70), thus proposing a channel-stabilizing PIP_2_ interaction with a region close to the pore. The C terminus of the Ca_V_2.1 α_1_-subunit has also been implicated in a PIP_2_-dependent regulation of the direct (and voltage-dependent) inhibition of Ca_V_2.1 channels by receptor-mediated G-protein activation. Evidence has been provided that this could be due to PIP_2_-mediated interaction with the plasma membrane because the C-terminal channel fragment showed binding to polyphosphoinositide strips *in vitro* (71). However, the polybasic cluster implicated in this interaction is not present in the C termini of LTCCs.

So far, we could not obtain evidence that the polybasic binding motif identified here is also important for the dynamic regulation of channel function by physiological modulatory pathways inducing phospholipid breakdown. Although we can show breakdown of PIP_2_ in our transfected cells by activation of M1 receptors robust enough to induce cytoplasmic relocation of the PIP_2_-selective probe PH-PLCδ, this did not result in relocation of our I-II linker constructs. In electrophysiological studies, Oxo-M stimulation of tsA-201 cells transfected with M1 receptors and Ca_V_1.2 induces a 33–60% inhibition of Ba^2+^ currents through Ca_V_1.2 (16), and more than half of this modulation is dependent on PIP_2_. This is compatible with models predicting that under these experimental conditions, 1–7% of the total PIP_2_ and 12–40% of PIP remain non-hydrolyzed in these cells (55). This may be sufficient for maintaining the binding of the I-II linker during stimulation with Oxo-M. Indeed, it is known from studies with proteins containing polybasic targeting motifs, similar to the one described here in the I-II linker, that efficient plasma membrane unbinding requires more than just PIP_2_ hydrolysis. The GTPases Rit and K-Ras require complete depletion of both PIP_2_ and PIP or PI3K products. Binding still persists when only one of them is depleted (22, 37). This is also in accordance with a previous study that observed that complete loss of the binding of the RasGRP1 plasma membrane targeting domain only occurred after combined treatment with the PLC activator m-3M3FBS and wortmannin and not with m-3M3FBS alone (56). In our experiments, dissociation of the Ca_V_1.2 I-II linker from the plasma membrane was also achieved by combining m-3M3FBS with ionomycin-induced Ca^2+^ increase. This suggests that Ca^2+^ also plays a role in this mechanism. Thus, it requires a second mechanism that either further enhances polyphosphoinositide breakdown (wortmannin) or a strong intracellular Ca^2+^ signal.

The requirement of Ca^2+^ could also indicate that PLCδ or phosphatidylserine plays a major role in reversing the plasma membrane targeting of the Ca_v_1.2 I-II linker. PLCδ is mainly activated by an increase in intracellular Ca^2+^ levels (10–100 nm), usually due to prior activation of other PLCs (72–74). Moreover, it has been shown that sustained Ca^2+^ increase can cause phosphatidylserine externalization (75). The observed translocation of the I-II linker upon m-3M3FBS and ionomycin application could therefore also be due to dissociation from the negatively charged phosphatidylserine. Hence, we propose that the I-II linker can interact with phosphoinositides as well as with phosphatidylserine, which has also been reported for RasGRP1 plasma membrane targeting (56). Independent of the exact mechanism, our biochemical studies provide evidence for a strong and constitutive binding of the polybasic membrane binding motif identified here, which may involve different negatively charged phospholipids.

The observation of facilitated gating and enhanced Ca^2+^ influx in our channel mutants is unexpected because recent work (76) has demonstrated inhibition of channel activity by M1 receptor-activated PIP_2_ breakdown. We were unable to test the effects of our mutations on M1 receptor-induced channel inhibition because the co-transfection was toxic for the cells and precluded their use in patch clamp recordings. Instead, we show that inducing phosphoinositide breakdown by treatment of cells with wortmannin and the PLC activator m-3M3FBS causes the expected inhibition of current amplitude but that this response is significantly attenuated by disruptive mutations in the polybasic motif as shown for Ca_v_1.2S_4E_ (Fig. 7). This further supports our interpretation that this membrane binding motif not only stabilizes a more reluctant gating mode but also supports moderation of channel activity by lipid breakdown once targeted to the plasma membrane.

The lipid-binding motif described here is positioned on the C-terminal end of the I-II linker. In contrast to the N-terminal half of the linker, this region has not previously been implicated in the control of Ca^2+^ channel function. The β-subunit tightly binds to a conserved motif within the N-terminal half of the I-II linker. X-ray crystallography and mutational studies (77–79) revealed that an α-helix between the AID (and its bound β-subunit) and IS6 provides a rigid connection to the channel pore and thereby crucially determines the voltage- and Ca^2+^-dependent gating properties of the channel. In Ca_V_2 channels, direct G-protein modulation is also mediated by Gβγ interaction in this region (77, 80). In contrast to this proximal region, no x-ray structure could be obtained C-terminal to the AID in a recent x-ray crystallographic study of the I-II linker in complex with β-subunit (77). Our data suggest that also this portion of the I-II linker forms an α-helix and that its interaction with membrane lipids plays a crucial role in stabilizing the channel conformation. This conformational stabilization may be transmitted either upstream through the I-II linker to the pore (IS6) or downstream through IIS1 to the voltage sensor of the repeat II, or both, and thereby control channel gating.

Based on recent genetic findings that already small changes in LTCC functions underlie human diseases (81), this polybasic motif may also be a target for human disease-causing mutations. Further studies must therefore investigate the consequences of single charge neutralizations within this motif as well as the role of adjacent positively charged residues for membrane binding and channel function.

## Author Contributions

G. K. and J. S. conceived the study. G. K. designed, performed, and analyzed the experiments shown in Figs. 1, 2, 3, and 5. A. P. designed, performed, and analyzed the experiments shown in Fig. 4. A. P., N. J. O., and A. L. designed, performed, and analyzed the experiments shown in Figs. 6 and 7. V. Y.-Y. designed, performed, and analyzed the experiments shown in Fig. 8. M. S.-B. supervised some of the cloning experiments. G. J. O. and B. E. F. supervised live cell imaging. G. K., A. P., and J. S. wrote the paper with input from all authors. J. S. coordinated the study. All authors critically evaluated the results and approved the final version of the manuscript.

## Footnotes

3 The abbreviations used are:

LTCC: L-type voltage-gated Ca^2+^ channel
AID: α-interaction domain
diC8-PIP_2_: phosphatidylinositiol 4,5-bisphosphate-diC8
*I*_Ca_: Ca^2+^ current
IP3: inositol-3-phosphate
*I*_Tail_: ionic tail current
*k*_act_: activation slope
LCI: live cell imaging
M1 receptor: muscarinic 1 receptor
Oxo-M: oxotremorine M
PIP: phosphatidylinositol 4-phosphate
PIP_2_: phosphatidylinositiol 4,5-bisphosphate
PIP_3_: phosphatidylinositiol 3,4,5-trisphosphate
PJ: pseudojanin
PLC: phospholipase C
Q_ON_: ON-gating charge movement
*V*_0.5_: half-maximal activation voltage
VGCC: voltage-gated Ca^2+^ channels
*V*_rev_: reversal potential
VSD: voltage sensor domain
PH: pleckstrin homology
EGFP: enhanced GFP
mRFP: monomeric RFP.

## References

[1] Catterall W. A. (2011) Voltage-gated calcium channels. Cold Spring Harb. Perspect. Biol. 3, a003947 21746798 10.1101/cshperspect.a003947 PMC3140680

[2] Striessnig J. Pinggera A. Kaur G. Bock G. Tuluc P. (2014) L-type calcium channels in heart and brain. Wiley Interdiscip. Rev. Membr. Transp. Signal. 3, 15–38 24683526 10.1002/wmts.102 PMC3968275

[3] Liao P. Yong T. F. Liang M. C. Yue D. T. Soong T. W. (2005) Splicing for alternative structures of Cav1.2 calcium channels in cardiac and smooth muscles. Cardiovasc. Res. 68, 197–203 16051206 10.1016/j.cardiores.2005.06.024

[4] Lipscombe D. Pan J. Q. Gray A. C. (2002) Functional diversity in neuronal voltage-gated calcium channels by alternative splicing of Cavα1. Mol. Neurobiol. 26, 21–44 12392054 10.1385/MN:26:1:021

[5] Lipscombe D. (2005) Neuronal proteins custom designed by alternative splicing. Curr. Opin. Neurobiol. 15, 358–363 15961039 10.1016/j.conb.2005.04.002

[6] Peterson B. Z. DeMaria C. D. Adelman J. P. Yue D. T. (1999) Calmodulin is the calcium sensor for calcium-dependent inactivation of L-type calcium channels. Neuron 22, 549–558 10197534 10.1016/s0896-6273(00)80709-6

[7] Grueter C. E. Abiria S. A. Dzhura I. Wu Y. Ham A. J. Mohler P. J. Anderson M. E. Colbran R. J. (2006) L-type Ca^2+^ channel facilitation mediated by phosphorylation of the β subunit by CaMKII. Mol. Cell 23, 641–650 16949361 10.1016/j.molcel.2006.07.006

[8] Hulme J. T. Westenbroek R. E. Scheuer T. Catterall W. A. (2006) Phosphorylation of serine 1928 in the distal C-terminal domain of cardiac Cav1.2 channels during β1-adrenergic regulation. Proc. Natl. Acad. Sci. U.S.A. 103, 16574–16579 17053072 10.1073/pnas.0607294103 PMC1637623

[9] Fu Y. Westenbroek R. E. Scheuer T. Catterall W. A. (2014) Basal and β-adrenergic regulation of the cardiac calcium channel Cav1.2 requires phosphorylation of serine 1700. Proc. Natl. Acad. Sci. U.S.A. 111, 16598–16603 25368181 10.1073/pnas.1419129111 PMC4246329

[10] Doering C. J. McRory J. E. (2007) Effects of extracellular pH on neuronal calcium channel activation. Neuroscience 146, 1032–1043 17434266 10.1016/j.neuroscience.2007.02.049

[11] De Waard M. Liu H. Walker D. Scott V. E. Gurnett C. A. Campbell K. P. (1997) Direct binding of G-protein βγ complex to voltage-dependent calcium channels. Nature 385, 446–450 9009193 10.1038/385446a0

[12] Roberts-Crowley M. L. Mitra-Ganguli T. Liu L. Rittenhouse A. R. (2009) Regulation of voltage-gated Ca^2+^ channels by lipids. Cell Calcium 45, 589–601 19419761 10.1016/j.ceca.2009.03.015 PMC2964877

[13] Suh B. C. Hille B. (2008) PIP2 is a necessary cofactor for ion channel function: how and why? Annu. Rev. Biophys. 37, 175–195 18573078 10.1146/annurev.biophys.37.032807.125859 PMC2692585

[14] Wu L. Bauer C. S. Zhen X. G. Xie C. Yang J. (2002) Dual regulation of voltage-gated calcium channels by PtdIns(4,5)P2. Nature 419, 947–952 12410316 10.1038/nature01118

[15] Gamper N. Reznikov V. Yamada Y. Yang J. Shapiro M. S. (2004) Phosphatidylinositol 4,5-bisphosphate signals underlie receptor-specific G_q/11_-mediated modulation of N-type Ca^2+^ channels. J. Neurosci. 24, 10980–10992 15574748 10.1523/JNEUROSCI.3869-04.2004 PMC6730206

[16] Suh B. C. Leal K. Hille B. (2010) Modulation of high-voltage activated Ca^2+^ channels by membrane phosphatidylinositol 4,5-bisphosphate. Neuron 67, 224–238 20670831 10.1016/j.neuron.2010.07.001 PMC2931829

[17] Suh B. C. Kim D. I. Falkenburger B. H. Hille B. (2012) Membrane-localized β-subunits alter the PIP2 regulation of high-voltage activated Ca^2+^ channels. Proc. Natl. Acad. Sci. U.S.A. 109, 3161–3166 22308488 10.1073/pnas.1121434109 PMC3287006

[18] Hermosilla T. Moreno C. Itfinca M. Altier C. Armisén R. Stutzin A. Zamponi G. W. Varela D. (2011) L-type calcium channel β subunit modulates angiotensin II responses in cardiomyocytes. Channels 5, 280–286 21525790 10.4161/chan.5.3.15833

[19] Roberts-Crowley M. L. Rittenhouse A. R. (2009) Arachidonic acid inhibition of L-type calcium (Cav1.3b) channels varies with accessory Cavβ subunits. J. Gen. Physiol. 133, 387–403 19332620 10.1085/jgp.200810047 PMC2699108

[20] Michailidis I. E. Zhang Y. Yang J. (2007) The lipid connection-regulation of voltage-gated Ca^2+^ channels by phosphoinositides. Pflugers Arch. 455, 147–155 17541627 10.1007/s00424-007-0272-9

[21] Yeung T. Gilbert G. E. Shi J. Silvius J. Kapus A. Grinstein S. (2008) Membrane phosphatidylserine regulates surface charge and protein localization. Science 319, 210–213 18187657 10.1126/science.1152066

[22] Heo W. D. Inoue T. Park W. S. Kim M. L. Park B. O. Wandless T. J. Meyer T. (2006) PI(3,4,5)P3 and PI(4,5)P2 lipids target proteins with polybasic clusters to the plasma membrane. Science 314, 1458–1461 17095657 10.1126/science.1134389 PMC3579512

[23] Hansen S. B. Tao X. MacKinnon R. (2011) Structural basis of PIP2 activation of the classical inward rectifier K^+^ channel Kir2.2. Nature 477, 495–498 21874019 10.1038/nature10370 PMC3324908

[24] Whorton M. R. MacKinnon R. (2011) Crystal structure of the mammalian GIRK2 K^+^ channel and gating regulation by G proteins, PIP2, and sodium. Cell 147, 199–208 21962516 10.1016/j.cell.2011.07.046 PMC3243363

[25] Hille B. Dickson E. J. Kruse M. Vivas O. Suh B. C. (2015) Phosphoinositides regulate ion channels. Biochim. Biophys. Acta 1851, 844–856 25241941 10.1016/j.bbalip.2014.09.010 PMC4364932

[26] Altier C. Garcia-Caballero A. Simms B. You H. Chen L. Walcher J. Tedford H. W. Hermosilla T. Zamponi G. W. (2011) The Cavβ subunit prevents RFP2-mediated ubiquitination and proteasomal degradation of L-type channels. Nat. Neurosci. 14, 173–180 21186355 10.1038/nn.2712

[27] Hilgemann D. W. (2012) Fitting K(V) potassium channels into the PIP(2) puzzle: Hille group connects dots between illustrious HH groups. J. Gen Physiol. 140, 245–248 22930801 10.1085/jgp.201210874 PMC3434100

[28] Horton R. M. Hunt H. D. Ho S. N. Pullen J. K. Pease L. R. (1989) Engineering hybrid genes without the use of restriction enzymes: gene splicing by overlap extension. Gene 77, 61–68 2744488 10.1016/0378-1119(89)90359-4

[29] Long J. Z. Lackan C. S. Hadjantonakis A. K. (2005) Genetic and spectrally distinct *in vivo* imaging: embryonic stem cells and mice with widespread expression of a monomeric red fluorescent protein. BMC Biotechnol. 5, 20 15996270 10.1186/1472-6750-5-20 PMC1192791

[30] Obermair G. J. Kugler G. Flucher B. E. (2004) The role of the calcium channel α2 δ-1 subunit in skeletal muscle. J. Muscle Res. Cell Motil. 25, 239–240 15467389 10.1023/b:jure.0000038361.47060.fe

[31] Obermair G. J. Schlick B. Di Biase V. Subramanyam P. Gebhart M. Baumgartner S. Flucher B. E. (2010) Reciprocal interactions regulate targeting of calcium channel β subunits and membrane expression of α_1_ subunits in cultured hippocampal neurons. J. Biol. Chem. 285, 5776–5791 19996312 10.1074/jbc.M109.044271 PMC2820804

[32] Dayal A. Bhat V. Franzini-Armstrong C. Grabner M. (2013) Domain cooperativity in the β1a subunit is essential for dihydropyridine receptor voltage sensing in skeletal muscle. Proc. Natl. Acad. Sci. U.S.A. 110, 7488–7493 23589859 10.1073/pnas.1301087110 PMC3645543

[33] Campiglio M. Di Biase V. Tuluc P. Flucher B. E. (2013) Stable incorporation versus dynamic exchange of β subunits in a native Ca^2+^ channel complex. J. Cell Sci. 126, 2092–2101 23447673 10.1242/jcs.jcs124537 PMC4148589

[34] van der Wal J. Habets R. Várnai P. Balla T. Jalink K. (2001) Monitoring agonist-induced phospholipase C activation in live cells by fluorescence resonance energy transfer. J. Biol. Chem. 276, 15337–15344 11152673 10.1074/jbc.M007194200

[35] Grabner M. Dirksen R. T. Beam K. G. (1998) Tagging with green fluorescent protein reveals a distinct subcellular distribution of L-type and non-L-type calcium channels expressed in dysgenic myotubes. Proc. Natl. Acad. Sci. U.S.A. 95, 1903–1908 9465115 10.1073/pnas.95.4.1903 PMC19211

[36] Lindner M. Leitner M. G. Halaszovich C. R. Hammond G. R. Oliver D. (2011) Probing the regulation of TASK potassium channels by PI4,5P(2) with switchable phosphoinositide phosphatases. J. Physiol. 589, 3149–3162 21540350 10.1113/jphysiol.2011.208983 PMC3145931

[37] Hammond G. R. Fischer M. J. Anderson K. E. Holdich J. Koteci A. Balla T. Irvine R. F. (2012) PI4P and PI(4,5)P2 are essential but independent lipid determinants of membrane identity. Science 337, 727–730 22722250 10.1126/science.1222483 PMC3646512

[38] Ortner N. J. Bock G. Vandael D. H. Mauersberger R. Draheim H. J. Gust R. Carbone E. Tuluc P. Striessnig J. (2014) Pyrimidine-2,4,6-triones are a new class of voltage-gated L-type Ca^2+^ channel activators. Nat. Commun. 5, 3897 24941892 10.1038/ncomms4897 PMC4083433

[39] Barth P. Schonbrun J. Baker D. (2007) Toward high-resolution prediction and design of transmembrane helical protein structures. Proc. Natl. Acad. Sci. U.S.A. 104, 15682–15687 17905872 10.1073/pnas.0702515104 PMC2000396

[40] Yarov-Yarovoy V. Schonbrun J. Baker D. (2006) Multipass membrane protein structure prediction using Rosetta. Proteins 62, 1010–1025 16372357 10.1002/prot.20817 PMC1479309

[41] Yarov-Yarovoy V. Baker D. Catterall W. A. (2006) Voltage sensor conformations in the open and closed states in ROSETTA structural models of K^+^ channels. Proc. Natl. Acad. Sci. U.S.A. 103, 7292–7297 16648251 10.1073/pnas.0602350103 PMC1464335

[42] Payandeh J. Scheuer T. Zheng N. Catterall W. A. (2011) The crystal structure of a voltage-gated sodium channel. Nature 475, 353–358 21743477 10.1038/nature10238 PMC3266868

[43] Hildebrand A. Remmert M. Biegert A. Söding J. (2009) Fast and accurate automatic structure prediction with HHpred. Proteins 77, 128–132 19626712 10.1002/prot.22499

[44] Söding J. Biegert A. Lupas A. N. (2005) The HHpred interactive server for protein homology detection and structure prediction. Nucleic Acids Res. 33, W244–W248 15980461 10.1093/nar/gki408 PMC1160169

[45] Wang C. Bradley P. Baker D. (2007) Protein-protein docking with backbone flexibility. J. Mol. Biol. 373, 503–519 17825317 10.1016/j.jmb.2007.07.050

[46] Yarov-Yarovoy V. DeCaen P. G. Westenbroek R. E. Pan C. Y. Scheuer T. Baker D. Catterall W. A. (2012) Structural basis for gating charge movement in the voltage sensor of a sodium channel. Proc. Natl. Acad. Sci. U.S.A. 109, E93–E102 22160714 10.1073/pnas.1118434109 PMC3258622

[47] Bonneau R. Strauss C. E. Rohl C. A. Chivian D. Bradley P. Malmström L. Robertson T. Baker D. (2002) *De novo* prediction of three-dimensional structures for major protein families. J. Mol. Biol. 322, 65–78 12215415 10.1016/s0022-2836(02)00698-8

[48] Pettersen E. F. Goddard T. D. Huang C. C. Couch G. S. Greenblatt D. M. Meng E. C. Ferrin T. E. (2004) UCSF Chimera: a visualization system for exploratory research and analysis. J. Comput. Chem. 25, 1605–1612 15264254 10.1002/jcc.20084

[49] Gebhart M. Juhasz-Vedres G. Zuccotti A. Brandt N. Engel J. Trockenbacher A. Kaur G. Obermair G. J. Knipper M. Koschak A. Striessnig J. (2010) Modulation of Cav1.3 Ca^2+^ channel gating by Rab3 interacting molecule. Mol. Cell Neurosci. 44, 246–259 20363327 10.1016/j.mcn.2010.03.011

[50] Takahashi S. X. Miriyala J. Tay L. H. Yue D. T. Colecraft H. M. (2005) A CaVbeta SH3/guanylate kinase domain interaction regulates multiple properties of voltage-gated Ca^2+^ channels. J. Gen. Physiol. 126, 365–377 16186563 10.1085/jgp.200509354 PMC2266626

[51] Van Petegem F. Clark K. A. Chatelain F. C. Minor D. L. Jr. (2004) Structure of a complex between a voltage-gated calcium channel β-subunit and an α-subunit domain. Nature 429, 671–675 15141227 10.1038/nature02588 PMC3076333

[52] Leroy J. Richards M. S. Butcher A. J. Nieto-Rostro M. Pratt W. S. Davies A. Dolphin A. C. (2005) Interaction via a key tryptophan in the I-II linker of N-type calcium channels is required for β1 but not for palmitoylated β2, implicating an additional binding site in the regulation of channel voltage-dependent properties. J. Neurosci. 25, 6984–6996 16049174 10.1523/JNEUROSCI.1137-05.2005 PMC6724838

[53] Buchan D. W. Ward S. M. Lobley A. E. Nugent T. C. Bryson K. Jones D. T. (2010) Protein annotation and modelling servers at University College London. Nucleic Acids Res. 38, W563–W568 20507913 10.1093/nar/gkq427 PMC2896093

[54] Kadamur G. Ross E. M. (2013) Mammalian phospholipase C. Annu. Rev. Physiol. 75, 127–154 23140367 10.1146/annurev-physiol-030212-183750

[55] Horowitz L. F. Hirdes W. Suh B. C. Hilgemann D. W. Mackie K. Hille B. (2005) Phospholipase C in living cells: activation, inhibition, Ca^2+^ requirement, and regulation of M current. J. Gen Physiol. 126, 243–262 16129772 10.1085/jgp.200509309 PMC2266577

[56] Zahedi B. Goo H. J. Beaulieu N. Tazmini G. Kay R. J. Cornell R. B. (2011) Phosphoinositide 3-kinase regulates plasma membrane targeting of the Ras-specific exchange factor RasGRP1. J. Biol. Chem. 286, 12712–12723 21285350 10.1074/jbc.M110.189605 PMC3069471

[57] Bae Y. S. Lee T. G. Park J. C. Hur J. H. Kim Y. Heo K. Kwak J. Y. Suh P. G. Ryu S. H. (2003) Identification of a compound that directly stimulates phospholipase C activity. Mol. Pharmacol. 63, 1043–1050 12695532 10.1124/mol.63.5.1043

[58] Várnai P. Balla T. (1998) Visualization of phosphoinositides that bind pleckstrin homology domains: calcium- and agonist-induced dynamic changes and relationship to myo-[^3^H]inositol-labeled phosphoinositide pools. J. Cell Biol. 143, 501–510 9786958 10.1083/jcb.143.2.501 PMC2132833

[59] Fuller M. D. Emrick M. A. Sadilek M. Scheuer T. Catterall W. A. (2010) Molecular mechanism of calcium channel regulation in the fight-or-flight response. Sci. Signal. 3, ra70 20876873 10.1126/scisignal.2001152 PMC3063709

[60] Hulme J. T. Konoki K. Lin T. W. Gritsenko M. A. Camp D. G. 2nd Bigelow D. J. Catterall W. A. (2005) Sites of proteolytic processing and noncovalent association of the distal C-terminal domain of Cav1.1 channels in skeletal muscle. Proc. Natl. Acad. Sci. U.S.A. 102, 5274–5279 15793008 10.1073/pnas.0409885102 PMC555994

[61] Hulme J. T. Yarov-Yarovoy V. Lin T. W. Scheuer T. Catterall W. A. (2006) Autoinhibitory control of the Cav1.2 channel by its proteolytically processed distal C-terminal domain. J. Physiol. 576, 87–102 16809371 10.1113/jphysiol.2006.111799 PMC1995633

[62] Lieb A. Scharinger A. Sartori S. Sinnegger-Brauns M. J. Striessnig J. (2012) Structural determinants of CaV1.3 L-type calcium channel gating. Channels 6, 197–205 22760075 10.4161/chan.21002 PMC3431584

[63] Singh A. Gebhart M. Fritsch R. Sinnegger-Brauns M. J. Poggiani C. Hoda J. C. Engel J. Romanin C. Striessnig J. Koschak A. (2008) Modulation of voltage- and Ca^2+^-dependent gating of Cav1.3 L-type calcium channels by alternative splicing of a C-terminal regulatory domain. J. Biol. Chem. 283, 20733–20744 18482979 10.1074/jbc.M802254200 PMC2475692

[64] Cao Y. Bartolomé-Martín D. Rotem N. Rozas C. Dellal S. S. Chacon M. A. Kadriu B. Gulinello M. Khodakhah K. Faber D. S. (2015) Rescue of homeostatic regulation of striatal excitability and locomotor activity in a mouse model of Huntington's disease. Proc. Natl. Acad. Sci. U.S.A. 112, 2239–2244 25646456 10.1073/pnas.1405748112 PMC4343133

[65] Hall D. D. Dai S. Tseng P.-Y. Malik Z. Nguyen M. Matt L. Schnizler K. Shephard A. Mohapatra D. P. Tsuruta F. Dolmetsch R. E. Christel C. J. Lee A. Burette A. Weinberg R. J. Hell J. W. (2013) Competition between α-actinin and Ca^2+^-calmodulin controls surface retention of the L-type Ca^2+^ channel Cav1.2. Neuron 78, 483–497 23664615 10.1016/j.neuron.2013.02.032 PMC4570828

[66] Rhee S. G. Choi K. D. (1992) Multiple forms of phospholipase C isozymes and their activation mechanisms. Adv. Second Messenger Phosphoprotein Res. 26, 35–61 1419362

[67] Miranda-Laferte E. Ewers D. Guzman R. E. Jordan N. Schmidt S. Hidalgo P. (2014) The N-terminal domain tethers the voltage-gated calcium channel β2e-subunit to the plasma membrane via electrostatic and hydrophobic interactions. J. Biol. Chem. 289, 10387–10398 24519939 10.1074/jbc.M113.507244 PMC4036161

[68] Gonzalez-Gutierrez G. Miranda-Laferte E. Contreras G. Neely A. Hidalgo P. (2010) Swapping the I-II intracellular linker between L-type Cav1.2 and R-type Cav2.3 high-voltage gated calcium channels exchanges activation attributes. Channels 4, 42–50 20026913 10.4161/chan.4.1.10562

[69] Neely A. Hidalgo P. (2014) Structure-function of proteins interacting with the α1 pore-forming subunit of high-voltage-activated calcium channels. Front. Physiol. 5, 209 24917826 10.3389/fphys.2014.00209 PMC4042065

[70] Zhen X. G. Xie C. Yamada Y. Zhang Y. Doyle C. Yang J. (2006) A single amino acid mutation attenuates rundown of voltage-gated calcium channels. FEBS Lett. 580, 5733–5738 17010345 10.1016/j.febslet.2006.09.027 PMC1693970

[71] Rousset M. Cens T. Gouin-Charnet A. Scamps F. Charnet P. (2004) Calcium and phosphatidylinositol 4,5-bisphosphate stabilize a Gβγ-sensitive state of Ca V2 Ca^2+^ channels. J. Biol. Chem. 279, 14619–14630 14722074 10.1074/jbc.M313284200

[72] Yang Y. R. Follo M. Y. Cocco L. Suh P. G. (2013) The physiological roles of primary phospholipase C. Adv. Biol. Regul. 53, 232–241 24041464 10.1016/j.jbior.2013.08.003

[73] Rhee S. G. Bae Y. S. (1997) Regulation of phosphoinositide-specific phospholipase C isozymes. J. Biol. Chem. 272, 15045–15048 9182519 10.1074/jbc.272.24.15045

[74] Suh P. G. Park J. I. Manzoli L. Cocco L. Peak J. C. Katan M. Fukami K. Kataoka T. Yun S. Ryu S. H. (2008) Multiple roles of phosphoinositide-specific phospholipase C isozymes. BMB Rep. 41, 415–434 18593525 10.5483/bmbrep.2008.41.6.415

[75] Balasubramanian K. Mirnikjoo B. Schroit A. J. (2007) Regulated externalization of phosphatidylserine at the cell surface: implications for apoptosis. J. Biol. Chem. 282, 18357–18364 17470427 10.1074/jbc.M700202200

[76] Suh B. C. Hille B. (2002) Recovery from muscarinic modulation of M current channels requires phosphatidylinositol 4,5-bisphosphate synthesis. Neuron 35, 507–520 12165472 10.1016/s0896-6273(02)00790-0

[77] Almagor L. Chomsky-Hecht O. Ben-Mocha A. Hendin-Barak D. Dascal N. Hirsch J. A. (2012) The role of a voltage-dependent Ca^2+^ channel intracellular linker: a structure-function analysis. J. Neurosci. 32, 7602–7613 22649239 10.1523/JNEUROSCI.5727-11.2012 PMC6703595

[78] Baumgart J. P. Vitko I. Bidaud I. Kondratskyi A. Lory P. Perez-Reyes E. (2008) I-II loop structural determinants in the gating and surface expression of low voltage-activated calcium channels. PLoS One 3, e2976 18714336 10.1371/journal.pone.0002976 PMC2495038

[79] Findeisen F. Minor D. L. Jr. (2009) Disruption of the IS6-AID linker affects voltage-gated calcium channel inactivation and facilitation. J. Gen. Physiol. 133, 327–343 19237593 10.1085/jgp.200810143 PMC2654080

[80] Tedford H. W. Kisilevsky A. E. Vieira L. B. Varela D. Chen L. Zamponi G. W. (2010) Scanning mutagenesis of the I-II loop of the Cav2.2 calcium channel identifies residues arginine 376 and valine 416 as molecular determinants of voltage dependent G protein inhibition. Mol. Brain 3, 6 20181083 10.1186/1756-6606-3-6 PMC2829547

[81] Pinggera A. Lieb A. Benedetti B. Lampert M. Monteleone S. Liedl K. R. Tuluc P. Striessnig J. (2015) CACNA1D *de novo* mutations in autism spectrum disorders activate Cav1.3 L-type calcium channels. Biol. Psychiatry 77, 816–822 25620733 10.1016/j.biopsych.2014.11.020 PMC4401440

